# A Virus-Like-Particle-Based Conjugate Vaccine Targeting the Microtubule Binding Region of Tau Protein

**DOI:** 10.1021/acsomega.6c00852

**Published:** 2026-07-15

**Authors:** Hunter McFall-Boegeman, Cameron Talbot, Mauro Montalbano, Nicha Puangmalai, Kuang-Wei Wang, Meena Kannan, Jiaming Shi, Katrina Linning-Duffy, Setare Nick, Lily Yan, Min-Hao Kuo, Rakez Kayed, Xuefei Huang

**Affiliations:** † Department of Chemistry, 3078 Michigan State University, East Lansing, Michigan 48824, United States; ‡ Institute for Quantitative Health Science and Engineering, Michigan State University, East Lansing, Michigan 48824, United States; § Mitchell Center for Neurodegenerative Diseases, University of Texas Medical Branch, Galveston, Texas 77555, United States; ∥ Departments of Neurology, Neuroscience and Cell Biology, University of Texas Medical Branch, Galveston, Texas 77555, United States; ⊥ Department of Biochemistry and Molecular Biology, Michigan State University, East Lansing, Michigan 48824, United States; # Department of Psychology, Michigan State University, East Lansing, Michigan 48824, United States; ∇ Department of Biomedical Engineering, Michigan State University, East Lansing, Michigan 48824, United States

## Abstract

There is a pressing need to develop novel strategies to ameliorate symptoms and slow the progression of Alzheimer’s disease. One of the hallmarks of Alzheimer’s disease is the high levels of tau protein, which can form toxic oligomers and characteristic neurofibrillary tangles in the brain. Antitau antibodies can potentially bind tau protein and reduce tau pathology. In order to elicit a powerful antitau antibody response, virus-like-particle bacteriophage Qβ-based conjugate vaccines were developed targeting the microtubule binding region of tau protein. The Qβ-tau vaccines were able to produce a strong antitau antibody response in not only wild-type mice but also in human tau transgenic mice and a llama. The levels of antibody induced were superior to those generated by a corresponding keyhole limpet hemocyanin-based tau conjugate mimicking the one vaccine that successfully completed phase 1/2 human clinical trials. The Qβ-tau vaccine significantly improved the cognitive functions of the immunized mice and reduced the levels of inflammatory cytokines and tau in the brains, suggesting its translational potential.

## Graphical abstract

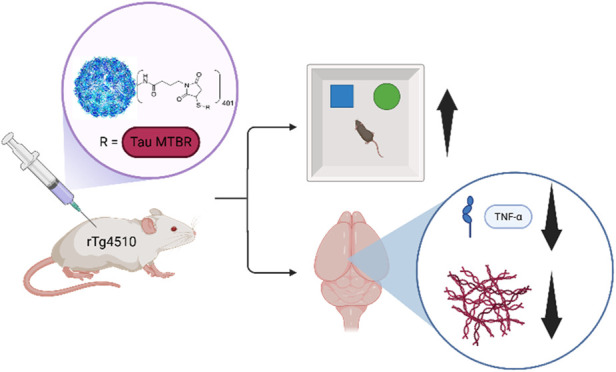

## Introduction

Alzheimer’s disease (AD) is one of the most common forms of dementia. It is characterized by progressive neurodegeneration resulting in loss of memory and cognitive abilities. Beyond the tremendous emotional and personal tolls associated with the disease, the patient care needed is estimated to cost $384 billion/year in the United States.^1^ These facts suggest an urgent need for effective treatments to ameliorate symptoms and slow the disease progression.

One of the hallmarks of AD is the buildup of neurofibrillary tangles (NFTs), with tau proteins being a major component.^2^ Tau is an intrinsically disordered protein that plays an important role in stabilizing microtubules in axons in healthy tissue. As part of the binding process to microtubules, physiological tau undergoes a conformational change, exposing a previously hidden hydrophobic region called the microtubule binding region (MTBR).^3,4^ In AD patients, tau has been found to be hyperphosphorylated.^5^ The increased charge density is believed to lead to conformational changes of pathological tau, which disrupt its binding to the microtubule and promote aggregation, possibly through exposure of hydrophobic regions, including the MTBRs.^6–8^ The exposed MTBRs can interact and oligomerize. Subsequently, tau aggregation can continue to increase, leading to toxic tau oligomers (tauO)^9–11^ and eventually NFTs (Figure 1A). Reduction of tau aggregate formation is a potential therapeutic strategy toward AD (Figure 1B).

**Figure 1:**
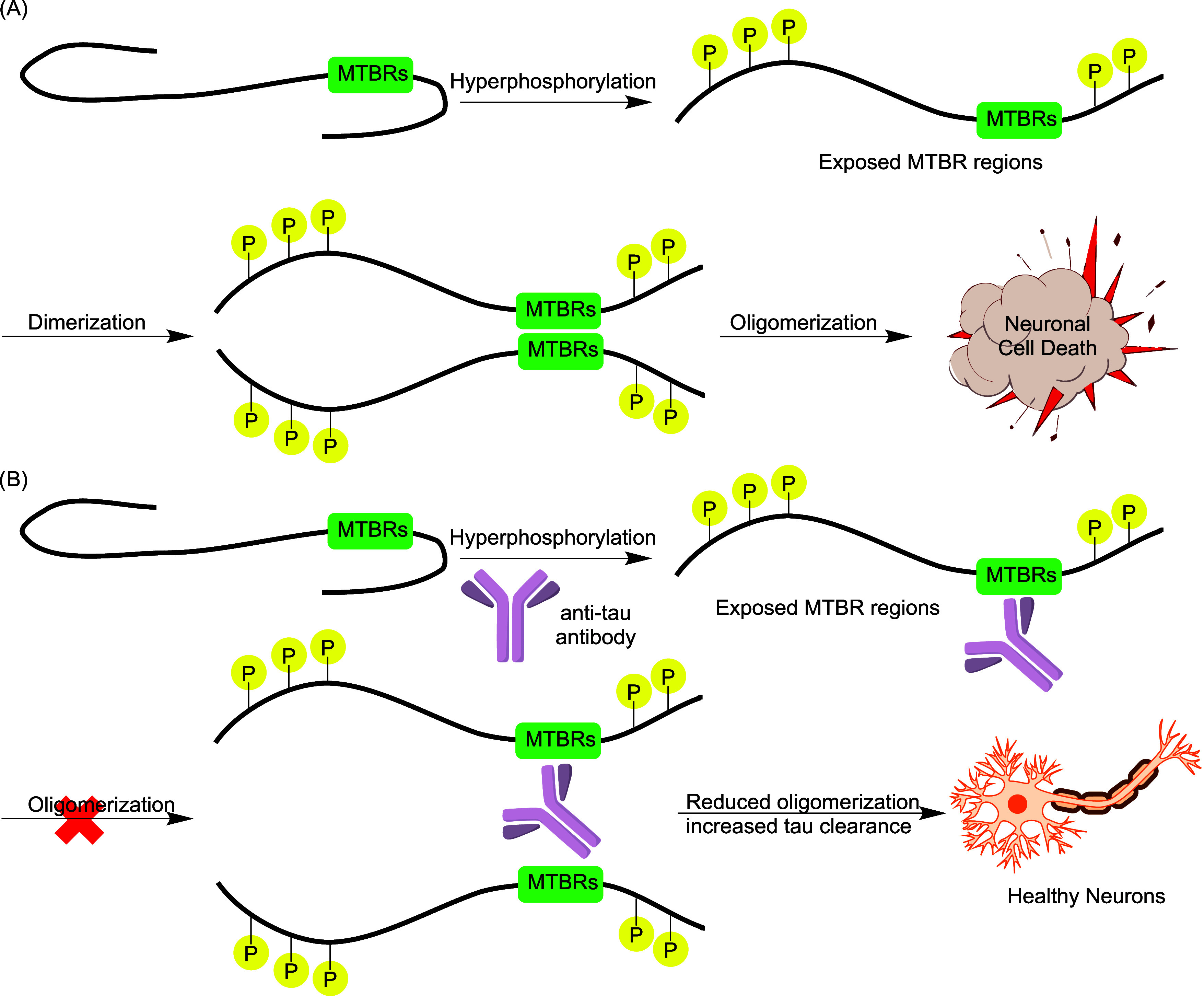
(A) In AD, hyperphosphorylation of tau leads to the exposure of MTBRs causing aggregation. Tau can continue to aggregate and oligomerize through interactions between MTBRs leading to neuronal cell death. (B) Antibodies generated against the MTBR region can potentially block MTBR interactions reducing oligomerization, increase the clearance of disease-associated tau, and reduce the adverse effects of pathogenic tau.

Immunotherapy that can generate antibodies against AD-associated pathogenic factors such as tau and amyloid β (Aβ) presents an appealing approach to treating the disease. Kontsekova and co-workers identified protective epitopes in tau’s MTBRs containing the minimal epitope sequence HXPGGG.^12^ This sequence is present in all four MTBR regions. Monoclonal antibodies recognizing these epitopes decreased tau oligomerization *in vitro* and *in vivo* ^12^ and can enhance the clearance of tau from the brain.^13^ Nasal delivery of antitau antibodies effectively reduced intracellular tau pathology (tauopathy) and improved cognitive functions in aged tauopathy mice.^14^ Complementary to antibody therapies, vaccines can potentially provide long-term protection against AD. A variety of tau-based vaccines have been studied,^15–22^ and hyperphosphorylated tau peptides have been investigated as the antigen for a vaccine, which reduced tauopathy in mice.^18,19,21^ The most advanced vaccine candidate, termed AADVac1, is a keyhole limpet hemocyanin (KLH) conjugate with the ^294^KDNIKHVPGGGS^305^ peptide sequence within human tau’s MTBRs, which has been evaluated in human phase I and II clinical trials, including patients with mild-to-moderate AD.^16,17,23–25^ The vaccine was shown to be immunogenic and safe in humans and slowed AD-related decline. Statistically significant inverse correlation was observed between the levels of total AADvac1-induced antitau antibodies and AD-associated symptoms. Thus, to enhance the efficacy of antitau vaccines, it is important to further improve the levels of antitau antibodies that can be produced for higher protection and therapeutic benefits.

The AADvac1 vaccine utilized KLH as a carrier in the conjugate vaccine. An alternative set of carriers in conjugate vaccine design is a virus-like particle (VLP). A potential advantage of VLPs over amorphous carriers such as KLH is their well-ordered structures, as highly organized antigen display can lead to superior antibody responses.^26^ A representative example of a VLP is the bacteriophage Qβ, which has been demonstrated to be a powerful vaccine carrier for a variety of antigens, including small molecule haptens, carbohydrates, and proteins.^27–32^ Recombinant expression of the coat protein (CP) of Qβ in *Escherichia coli* results in spontaneous assembly of the virus-like particle (VLP) composed of 180 copies of the CP in an icosahedral geometry.^33,34^ Conjugation of Qβ VLP with haptens can lead to helper T cell responses^35^ and significantly enhance immune responses toward the antigen as compared with the free antigen on its own.^29,31,36,37^ Additional improvements of the VLP Qβ have been realized by engineering a triple mutant of Qβ (mQβ) to reduce anticarrier antibodies while further enhancing the levels of antibodies produced against the hapten.^38^

Building on the promise of AADvac1 targeting the MTBR epitope in tau using the gold-standard carrier KLH and the potentially superior properties of Qβ as carrier proteins in conjugate vaccines, herein we report the ability of the VLP bacteriophage Qβ conjugated with the tau MTBR epitope (Qβ-tau) to elicit high titers of antitau antibodies. The antibodies induced were capable of recognizing the pathogenic tau *in vitro* as well as *ex vivo* in AD patient brains. Furthermore, use of the engineered mQβ conjugated with the tau MTBR epitope (mQβ-tau) further increased titers, and immunization of human tau transgenic mice with the mQβ-tau vaccine reduced AD-associated symptoms and increased cognitive abilities of the mice.

## Results and Discussion

### Synthesis and Characterization of Vaccine Constructs

For the tau vaccine study, we functionalized the surface lysines of Qβ for antigen conjugation. Qβ was first incubated with the bifunctional linker 4-maleimidobutyric acid *N*-hydroxysuccinimide ester (GMBS) (Scheme 1A). Following centrifuge filtration removing unreacted GMBS, the peptide CKDNIKHVPGGGS (tau_294–305_ with an additional cysteine added at its *N*-terminus) was conjugated to Qβ through the maleimide moiety on the linker. Mass spectrometry analysis showed that Qβ-tau had an average loading of 2.2 antigens per CP corresponding to 401 antigens/particle (Figure S1). As a positive control, KLH-tau was synthesized as previously reported through the GMBS linker to mimic AADvac1 (Scheme 1B).^12,23^ In addition, bovine serum albumin (BSA) conjugate of tau peptide (BSA-tau) was prepared for enzyme-linked immunosorbent assay (ELISA) analysis of the levels of antitau antibodies generated by the vaccine without the interference from antibodies against the carrier protein (Figure S2).

**Scheme 1:**
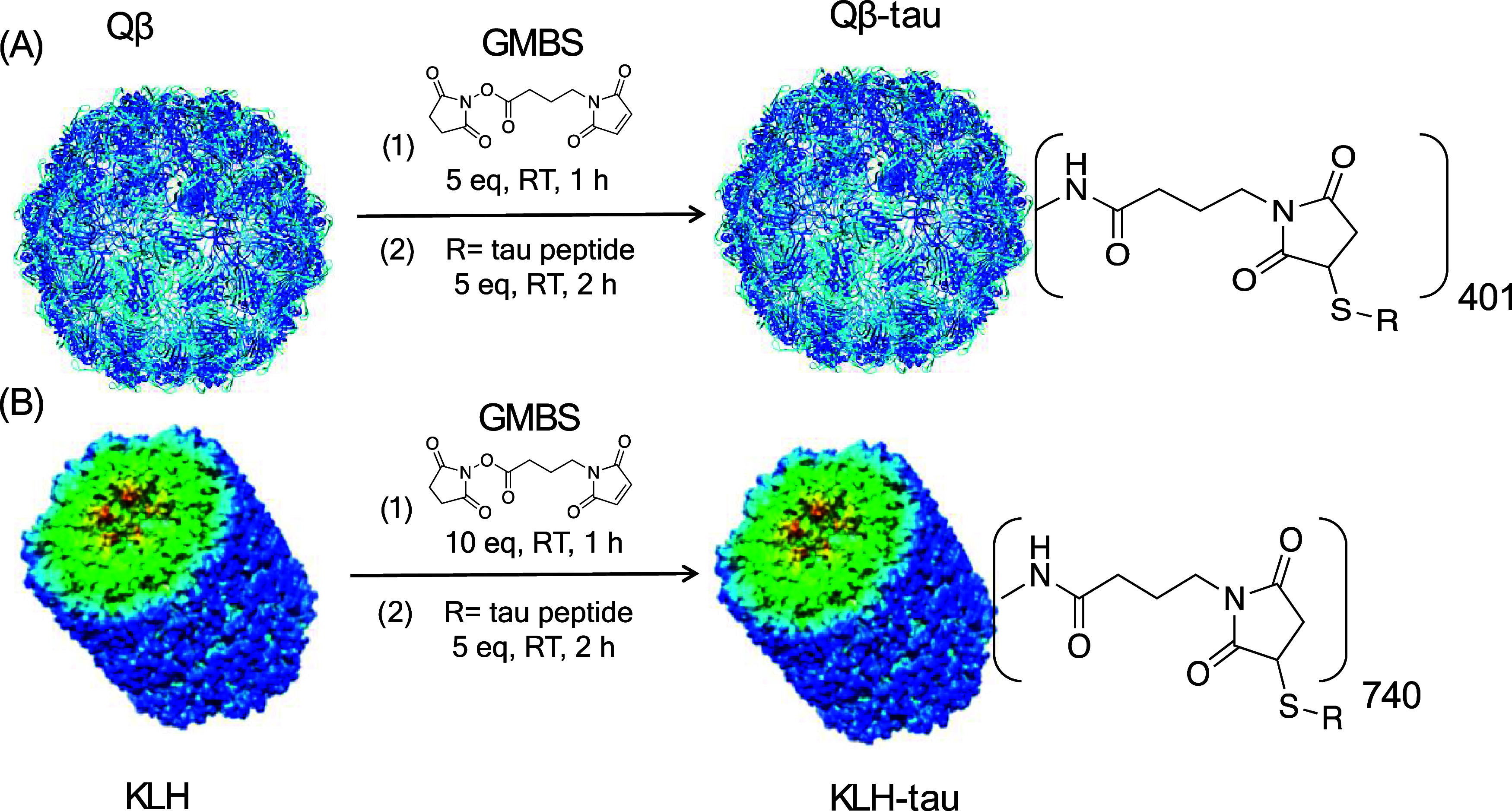
(A) Reaction Scheme for the Synthesis of Qβ-Tau Conjugate (EQ: Equivalent; RT: Room Temperature); The Average Loading Was 401 Copies of the Peptide Per Qβ Particle as Determined by Electrospray Ionization Mass Spectrometry; (B) Reaction Scheme for the Synthesis of KLH-Tau Peptide Conjugate

### Vaccination with Qβ-Tau Induced Long-Lasting Antitau Antibody Responses

With vaccine constructs in hand, we investigated the ability of the vaccine to elicit immune responses against tau. C57BL/6 mice were immunized with either Qβ-tau or KLH-tau (9.6 nmol or 12.5 μg of antigen, *N* = 5 for each group) with Alum (1 mg) as the adjuvant, with one prime and two boost injections spaced 2 weeks apart using the same formulation. In addition, another group (*n* = 5) of mice was administered phosphate-buffered saline (PBS) with Alum adjuvant as a control. Blood was drawn from the immunized mice for analysis of the levels of antibodies induced.

Mice immunized with Qβ-tau elicited super-high titers of IgG antibodies as determined through ELISA with BSA-tau as the coating antigen (Figure 2A). On day 21 after the prime immunization (1 week after the first booster injection), an average of 1 million ELISA units of antitau IgG antibodies was observed in Qβ-tau vaccinated mice. In contrast, the KLH-tau vaccinated group gave an average antitau IgG titer of 75,000 ELISA units (Figure 2A). The titers of antibodies in Qβ-tau vaccinated mice remained high over 90 days. The higher titers induced by Qβ-tau highlighted the superiority of Qβ as an immunogenic carrier as compared to the gold standard KLH. The subtypes of antibodies induced by Qβ-tau were analyzed. All subtypes of IgG antibodies, i.e., IgG1, IgG2b, IgG2c, and IgG3, were produced by immunization (Figure 2B), suggesting the activation of helper T cells through vaccination and the induction of antibody isotype switching to IgG.

**Figure 2:**
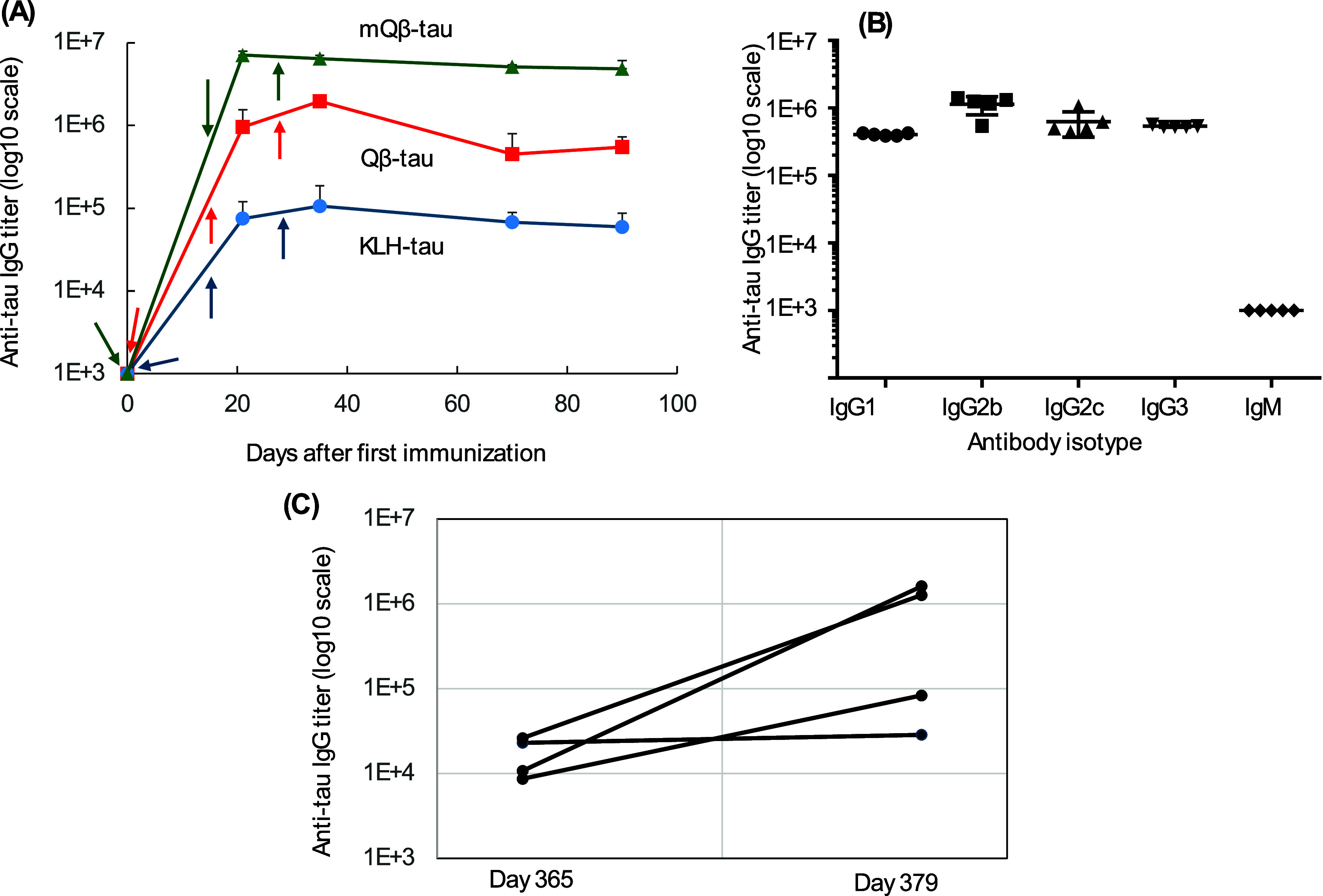
Immunization with Qβ-tau produced superior levels of antitau IgG titers in C57BL/6 mice as compared to immunization with KLH-tau. The antibodies elicited were mainly IgGs, and mice responded to booster injections. (A) Longitudinal antitau IgG titers from mice (*N* = 5) immunized with mQβ-tau, Qβ-tau, or KLH-tau on days 0, 14, and 28 as indicated by the arrows. (B) Immunoglobulin subtyping of day 35 sera from mice immunized with Qβ-tau. Each symbol represents one mouse in the group. The horizon bar represents the median titer of the group, with the error bar indicating standard deviation. (c) Antitau IgG titers from mice (*N* = 4) immunized with Qβ-tau on days 365 and 379 that received another booster on day 365. Individual mouse is represented by a symbol with the line connecting the responses on days 365 and 379 of each mouse. Titers were determined as the fold of dilution that gave an absorbance value above the average +3*SD of the blank. All measurements were made in quadruplicate.

As AD is a long-term disease, it is important to ensure that if the immune response wanes, it can be boosted. To this end, sera from immunized mice were collected on day 365 after the initial immunization, and mice were given a booster of the Qβ-tau vaccine on the same day. Two weeks later (day 379) sera were collected again, and pre- and postlast booster titers were compared (Figure 2C). Three out of the four mice responded to the boost, with two returning to titer levels near their day 35 peak. This suggests that immunization with Qβ-tau could generate long-lived memory B cells in some mice that were reactivated following an additional booster.

### Antibodies Induced by Qβ-Tau Are Capable of Recognizing Monomeric, Oligomeric, and Hyperphosphorylated Tau

With the knowledge that sera from immunized mice could recognize the tau peptide used for immunization, we next investigated whether the postimmune sera could bind tau proteins. We tested the ability of the induced antibodies to recognize the presumed toxic form of tau, i.e., tau oligomers (tauO),^39^ and monomeric tau. Western blotting demonstrated that antibodies from control mice immunized with Qβ did not stain strongly tauO or monomeric tau (Figure 3A). In contrast, sera from Qβ-tau-immunized mice recognized tauO and the tau monomer (Figure 3B), which is similar to those by a commercial pan-tau antibody (Tau5) (Figure 3C,D). The immune sera did not recognize another Alzheimer’s disease-associated protein Aβ42 (Figure 3). These results suggest that antibodies generated by Qβ-tau could bind not only the immunizing peptide antigen but also tau proteins, and the antibodies have high selectivity toward tau over Aβ.

**Figure 3:**
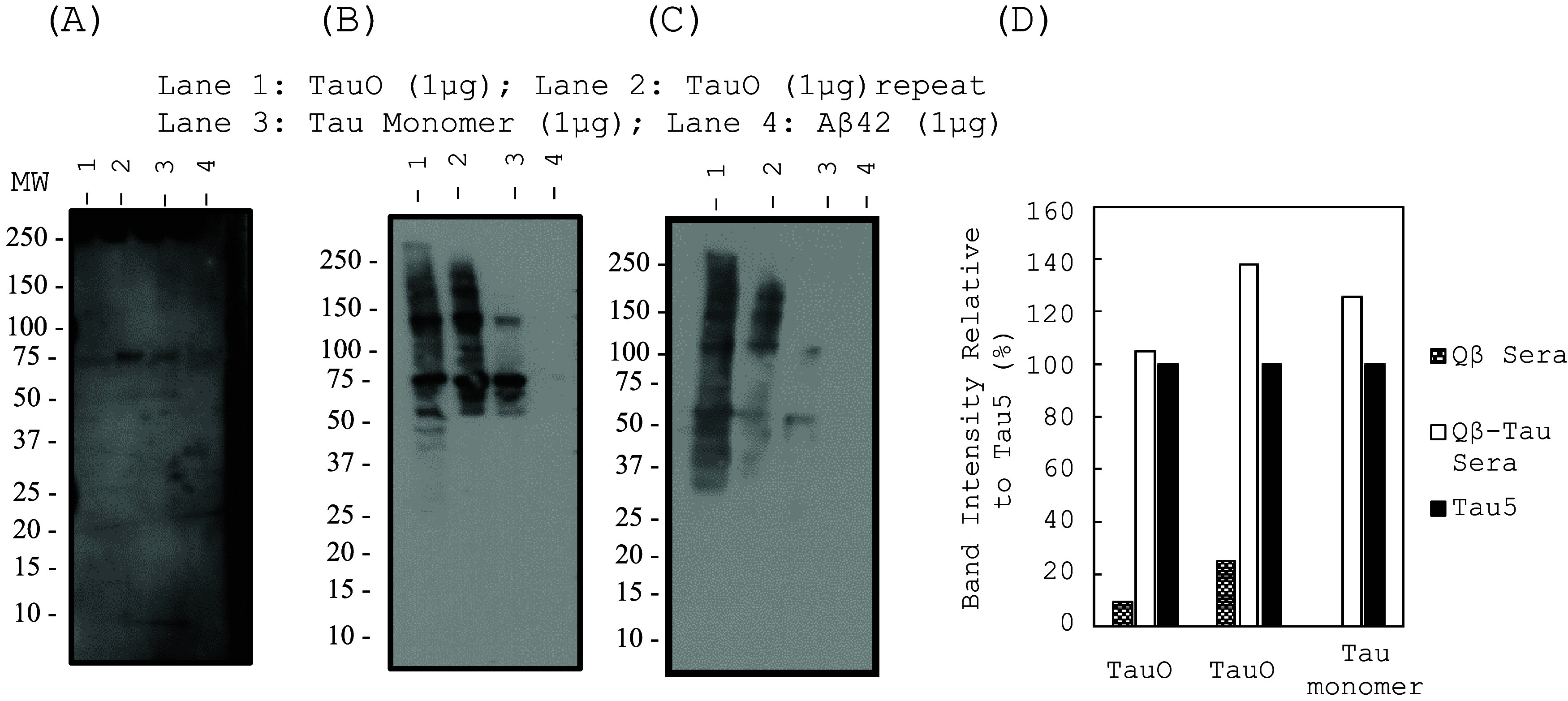
Western Blots against various recombinantly expressed tau constructs and Aβ42 (used as a negative control) by postimmune sera. (A) Sera from mice immunized with Qβ only were unable to recognize oligomeric tau (tauO) or monomeric tau. Lanes 1 and 2 are two different batches of tauO. Lane 3 is the tau monomer. Lane 4 is Aβ42. (B) Sera from mice immunized with Qβ-tau recognized tauO (lanes 1 and 2), tau monomer (lane 3), but not Aβ42 (lane 4). (C) A commercial pan-tau antibody (Tau5) recognized tauO (lanes 1 and 2), tau monomer (lane 3), but not Aβ42 (lane 4). (D) Quantification of band intensities of various tau constructs stained with different antibodies/sera as compared to Tau5 staining. The intensity of Tau5 staining on each gel was set as 100%. Serum was used at a 1:500 dilution. Tau5 (10 μg) was used at a 1:1000 dilution. Goat antimouse-HRP was used as the secondary antibody at a 1:1000 dilution.

We next examined the ability of the induced antibodies to recognize different forms of tau, including tau_151–391_,^40,41^ hyperphosphorylated tau (p-tau) based on the 1N4R isoform,^42^ and the full-length 2N4R tau with two *N*-terminal inserts (2N) and four microtubule binding repeats (4R). Titers of antibodies against various tau forms in day 35 sera were measured, with significant IgG titers generated against these forms of tau (Figure 4). Tau_151–391_ is a truncated version of the 2N4R isoform with four copies of the minimal epitope highly prone to aggregation. The tau_151–391_ construct is a disease-relevant model because of its ability to initiate tau aggregation.^40,41^ One of the theories underlying this model is that with the truncated *N-* and *C-*termini of tau_151–391_, its MTBR regions are exposed, which could be readily recognized by antibodies. For the p-tau tested, although it does not contain phosphorylation in the MTBR epitopes,^42^ its MTBR region may be more shielded compared to tau_151–391_, leading to lower titers. Among the three forms of tau tested, the unphosphorylated full-length 2N4R tau had the most variable responses, presumably because its MTBR region is shielded to varying degrees due to inhomogeneous aggregation.

**Figure 4:**
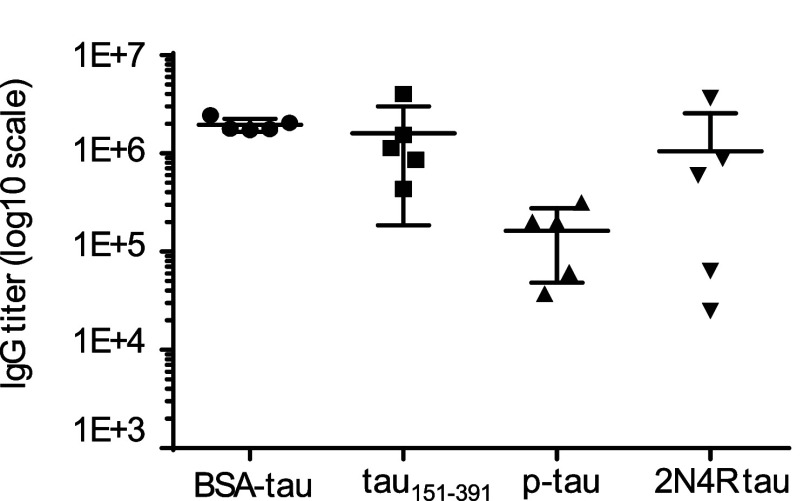
IgG titers from day 35 sera against various tau constructs. Individual sera (*N* = 5) from day 35 mice immunized with Qβ-tau. ELISA plates were coated with the antigen indicated on the *x*-axis. Titer values were calculated as the value that gave a signal greater than ave. + 3*SD of the blank. Each symbol represents one mouse in the group. The horizon bar represents the median titer of the group, with the error bar indicating standard deviation.

### Postimmune Sera Recognized Tau in Alzheimer’s Disease Patient Brain Tissues

Next, we tested whether the elicited antibodies were capable of staining tau in human tissues. Immunohistochemistry (IHC) was performed on brain tissue samples from AD patients (Figure 5). Slides were stained with Qβ-immunized mouse serum (negative control), Qβ-tau-immunized mouse serum, or a total tau monoclonal antibody (Tau5, positive control). Qβ-immunized serum showed little to no staining, whereas serum from Qβ-tau-immunized mice stained the tissues. None of the sera stained the brain tissues from a healthy control, suggesting the selectivity of the Qβ-tau-immunized serum toward AD tissues. While Tau5 is known to recognize the tau amino acid sequence 218–225 present in many tau species,^43^ it stained the brain tissue from an AD patient but not that from a normal control, reflecting higher levels of tau in the AD patient.

**Figure 5:**
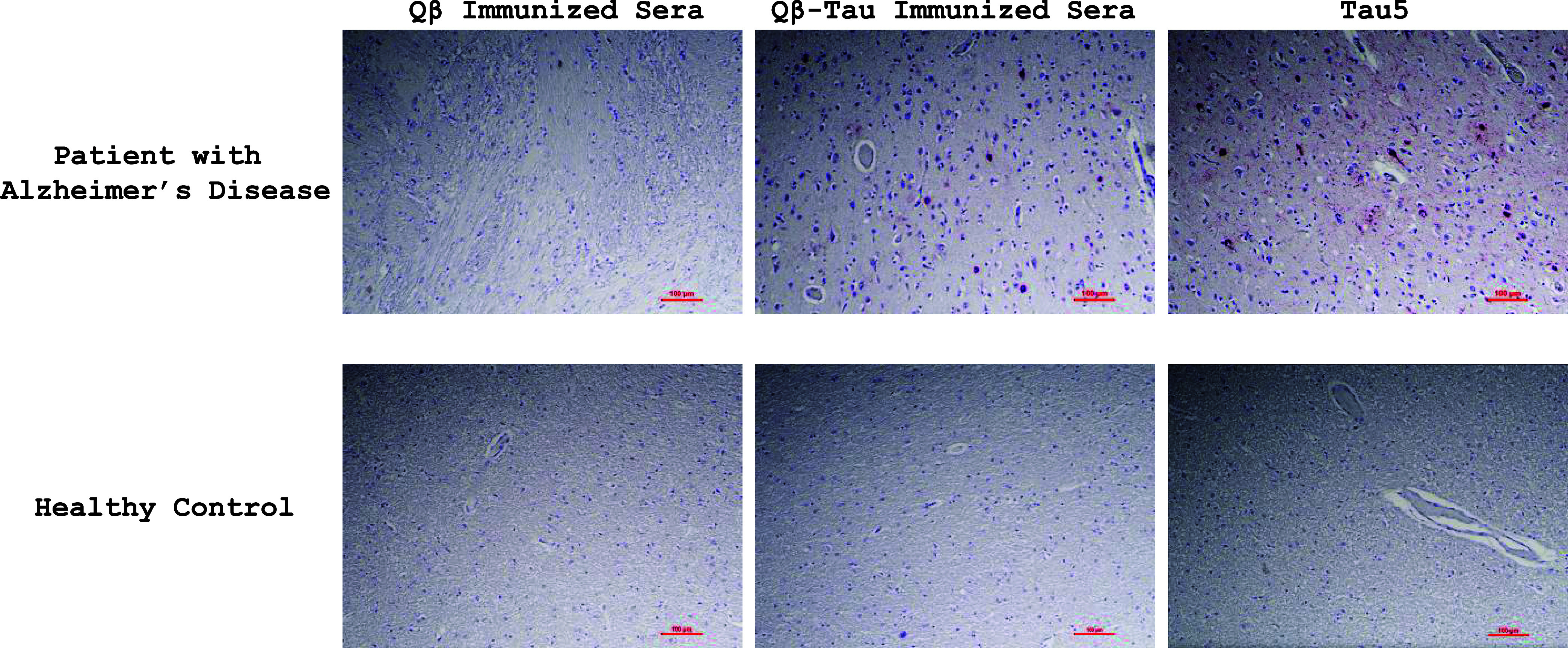
IHC of human brain tissue from a patient with Alzheimer’s disease or a healthy control. Slides were stained with day 35 pooled sera from mice immunized with Qβ, day-35 pooled sera from mice immunized with Qβ-tau, or the pan-tau monoclonal antibody Tau5. Sera from Qβ-tau-immunized mice showed a similar staining pattern to that of Tau5 in AD tissue and no staining in healthy tissue. The scale bars are 100 μm.

To test whether the elicited sera detect a similar tau population as that by the commercial antibodies, immunofluorescence (IF) was performed. IF staining of brains from patients with Alzheimer’s disease showed similar punctate staining and excellent correlation between Qβ-tau-immunized serum and total tau antibodies (Figure 6), suggesting that the sera were selective toward tau. As a negative control, healthy age and gender matched controls were also tested, which resulted in little staining by either Qβ-tau-immunized serum or the polyclonal total tau antibodies.

**Figure 6:**
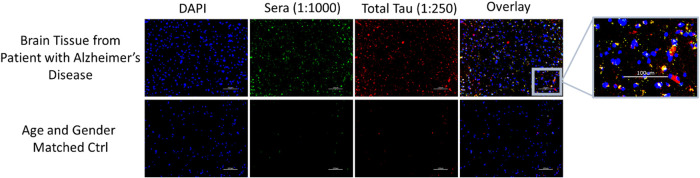
Immunofluorescence staining of brain tissue from either an AD patient or a healthy age and gender matched control. Tissue was stained with DAPI (blue color), day 365 pooled sera from mice immunized with Qβ-tau (green), or rabbit total tau antibodies (red, positive control). To visualize the staining, serum antibodies were stained with either goat-antimouse-Alexa Fluor 488 (for Qβ-tau-immunized mouse sera) or goat-antirabbit-Alexa Fluor 568 (for commercial total tau antibody from rabbits). The signals from Qβ-tau-immunized mouse sera and the positive control rabbit total tau antibodies tended to overlap in diseased tissues and showed minimal staining in healthy tissues. The scale bars are 100 μm.

### Examination of a Qβ Mutant as an Improved Carrier

One potential drawback of conjugate vaccines is that high antibody responses may be generated against the protein carrier, which can reduce the levels of antibodies produced against the target antigen.^44–46^ We have reported a Qβ mutant (mQβ) with the mutations of A38K/A40C/D102C, which is associated with reduced anticarrier antibody responses and increased the stability of the capsid.^38^ To test whether this mQβ carrier can further enhance antitau antibody levels, an mQβ-tau vaccine (Figure S3) was synthesized in a similar manner as the Qβ-tau construct with the GMBS linker. C57Bl6 mice were immunized with mQβ-tau following the identical immunization protocol and vaccine formulation as in the Qβ-tau study. Comparison of longitudinal titer data of C57BL/6 mice immunized with mQβ-tau showed higher antitau IgG titers at all times measured as compared to mice immunized with Qβ-tau (Figure 1A). Additionally, there were approximately 4 times fewer anticarrier antibodies elicited when using mQβ vs wild-type Qβ as the carrier (Figure S4). Mice immunized with mQβ-tau had an average antitau IgG titer of 7.1 million ELISA units on day 21 after the prime immunization, which was over 80 times higher than that from KLH-tau-immunized mice. Taken together, these data support the enhanced immunostimulatory effect against the target antigen from mQβ carrier.

### mQβ-Tau Vaccine Provided Protection to Mice against the Harmful Effects of Tau

To evaluate the potential protective efficacy of the mQβ-tau vaccine *in vivo*, human tau transgenic mice (rTg4510) were utilized. Unlike wild-type mice, rTg4510 mice express human tau in the forebrain and are prone to tauopathy.^47^ These mice provide a good translational model to evaluate a tau-targeting vaccine since they express human tau endogenously. They start to display cognitive and behavioral symptoms at ∼10 weeks of age^48^ with more pronounced cognitive and behavioral deficits at ∼16 weeks.^49^

rTg4510 mice were immunized with mQβ-tau, admixture of tau and mQβ, or PBS (*N* = 10 mice per group, 8 weeks old) following the aforementioned one prime two boost schedule (9.6 nmol peptide per mouse per injection) over 4 weeks with Alum as the adjuvant (Scheme S1). The admixture of tau and mQβ group was utilized as a comparison to establish the importance of the covalent linkage between mQβ and tau for antibody induction. Despite the fact that human tau is expressed endogenously in rTg4510 mice, 35 days after initial immunization with mQβ-tau, mice generated high levels of antitau IgG antibody responses with an average IgG titer of 5.1 million ELISA units, which were similar to those in mQβ-tau-immunized wild-type C57BL/6 mice (Figure 7). Control rTg4510 mice receiving an admixture of tau and Qβ or PBS only gave an average antitau IgG titer below 10,000 ELISA units.

**Figure 7:**
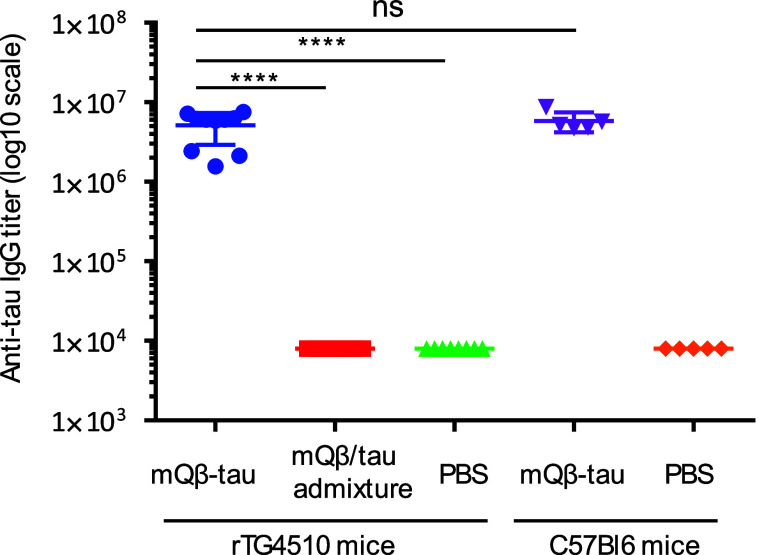
Immunization of the rTG4510 mice with mQβ-tau induced significantly higher antitau IgG antibody responses as compared to mice immunized with mQβ/tau admixture or PBS. Titer values were calculated as the value that gave a signal greater than ave.+3*SD of the blank. The starting serum dilution for ELISA was 16,000-folds, where sera from mice immunized with mQβ/tau admixture or PBS all showed background levels of signals. Thus, the titers for those groups were reported as 8,000 ELISA units. The antitau IgG antibody levels induced in rTG4510 mice were similar to those in mQβ-tau-immunized wild-type C57BL/6 mice. Each symbol represents one mouse in the group. The horizon bar represents the median titer of the group with the error bar indicating standard deviation. One-way ANOVA allowed for the rejection of the null hypothesis that all groups have the same levels of IgG titers (*p* < 0.0001). Statistical significance was performed by Tukey’s multiple comparisons posthoc test. ns: *p* > 0.05; **** *p* < 0.0001.

To assess the impact of the vaccine on the cognitive function of mice, the three groups of mice (mQβ-tau, admixture of mQβ and tau, and PBS) were subject to object memory task testing (Scheme S1). One known adverse effect of AD development is the loss of learning and memory.^47^ Memory impairments can be assessed in mice using the novel object recognition test, in which a mouse distinguishes between a novel object and a familiar one it has previously encountered.^50^ As shown in Figure 8, although the rTG4510 mice receiving PBS have a slight preference for the novel object as a cohort (an average of 60.4%), 5 out of the 10 animals performed near the chance level (a complete lack of preference would give a 50% chance in the novel object side), demonstrating no discrimination between the two objects and their impaired memory. In contrast, the mQβ-tau-immunized mouse group exhibited a significantly higher preference (an average of 80.1%) for exploring the novel object, with only 1 out of the 10 performing at the chance level. The superior memory performance in this group indicates a protective effect from vaccination. This improvement may be associated with the direct binding of tau by antibodies. Other immune-mediated mechanisms such as Fcγ receptor-mediated cellular uptake and lysosomal degradation may also contribute to cognitive benefits.^51^ The observed effects of the vaccine are consistent with tau immunotherapy that has been shown to reduce tau pathology and improve cognitive performance in tauopathy models, including Tg4510 mice.^52,53^

**Figure 8:**
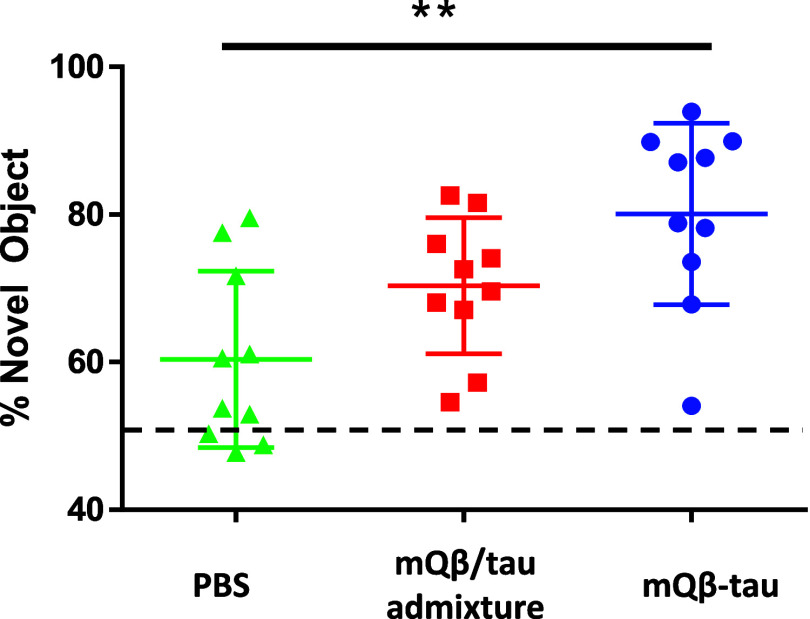
mQβ-tau immunization improved the cognitive function of rTg4510 mice as reflected by the significantly higher % of mQβ-tau-immunized mice in the side with novel objects as compared to those receiving PBS only. The horizontal dashed line at 50% indicates chance-level performance. One-way ANOVA allowed for the rejection of the null hypothesis that all groups have the same levels of preference for a novel object (*p* = 0.0023). Statistical significance was performed by Tukey’s multiple comparisons posthoc test. ** *p* < 0.01. Data are presented as mean ± SEM with data points from individual mice plotted (*n* = 10 per group).

A prominent characteristic of AD patients is the higher levels of soluble tau and tau deposits in the brain.^54^ To better understand the effects of vaccines on AD-like development in rTg4510 mice, their brains were collected. The brain tissues were homogenized, treated with sarkosyl detergent, and divided into sarkosyl-soluble and insoluble fractions. The levels of tau proteins in both fractions of the three groups of mice (mQβ-tau, admixture of tau and Qβ, and PBS) were quantified by Western blots (Figures 9, S9–S14). Compared with mice receiving PBS only or the admixture of tau and mQβ, mice immunized with mQβ-tau had significantly lower levels of tau in both the sarkosyl-soluble and insoluble fractions. It is believed that the insoluble tau deposit may be in equilibrium with soluble tau. Antibody binding and clearance of soluble tau may presumably lead to the dissolution of insoluble tau and enhance the removal of both soluble and insoluble tau from the brains of mQβ-tau-immunized mice.

**Figure 9:**
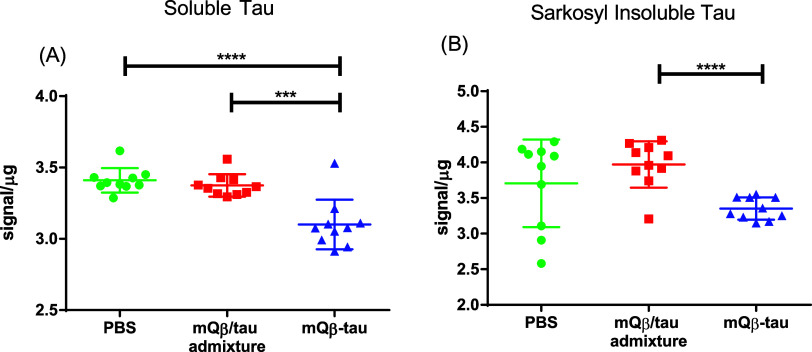
Quantification of the amounts of tau protein in (A) sarkosyl-soluble and (B) sarkosyl-insoluble fractions of the dorsal hippocampal subregion of mice immunized with mQβ-tau, mQβ/tau admixture, and PBS, respectively, via Western blot. Mice immunized with mQβ-tau had significantly lower levels of tau in both sarkosyl soluble and insoluble fractions. The *Y*-axis is the intensity of the Western blot band per μg of brain tissue. One-way ANOVA allowed for rejection of the null hypothesis that all groups have the same levels of tau (*p* = 0.0005 for panel A and *p* = 0.0052 for panel B). Statistical significance was performed by Tukey’s multiple comparisons posthoc test. ** *p* < 0.01, *** *p* < 0.001. Data are presented as mean ± SEM with data points from individual mice plotted (*n* = 10 per group).

Besides high tau levels, another common feature of Alzheimer’s disease is the increased neuroinflammation in the brain.^55,56^ Reduction of inflammation may potentially alleviate the symptoms and slow down disease progression. To analyze the vaccine effect on inflammation, the levels of proinflammatory cytokines in the dorsal hippocampal region of mouse brains were quantified by qPCR (Figure 10). mQβ-tau immunization significantly reduced the levels of inflammatory cytokines, including both interleukin-6 (IL-6) and tumor necrosis factor-α (TNF-α).

**Figure 10:**
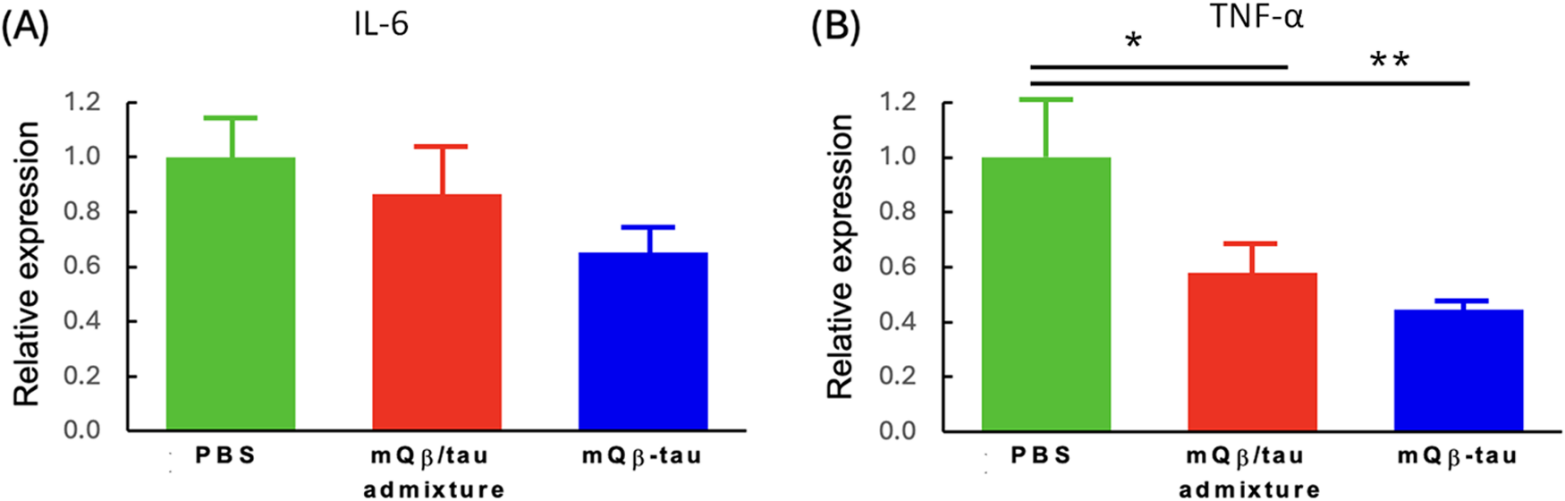
Quantification of the amounts of (A) IL-6 and (B) TNF-α in the dorsal hippocampal subregion of mice immunized with PBS, mQβ/tau admixture, and mQβ-tau, respectively. Mice immunized with mQβ-tau had significantly lower levels of IL-6 and TNF-α. One-way ANOVA allowed for the rejection of the null hypothesis that all groups have the same levels of TNF-α (*p* = 0.0017). Statistical significance was performed by Tukey’s multiple comparisons posthoc test. * *p* < 0.05, ** *p* < 0.01.

### mQβ-Tau Vaccine Was Immunogenic in Llama

As a step toward future translation, we performed an immunogenicity study of the mQβ-tau vaccine in a large animal model, a llama. The llama was immunized with mQβ-tau vaccine on days 0, 21, 49, and 77. Blood was collected preimmunization and on days 28, 56, and 84. mQβ-tau elicited significant antitau IgG antibodies as compared to preimmune sera. The major antitau IgG isotype induced was IgG1 (Figure S5). For IgG2/3, while there was an increase in antibody levels on day 84 as compared to preimmune, days 21, and 49 sera, the overall levels were low, presumably due to the small size of the tau peptide epitope. Llama IgG2/3 antibodies are known as nanobodies, which contain half of the complementarity-determining region loops as traditional IgG antibodies.^57^ This results in a smaller paratope surface area, which may have low affinities to a short linear peptide epitope such as the MTBR peptide.^58^ To induce higher levels of IgG2/3, further boosting with the vaccine may be needed.

## Conclusions

Qβ conjugate vaccines have been developed to target the MTBR region of tau. The mQβ-tau elicited over 80 times higher titers of antitau IgG antibodies on day 21 postimmunization than the corresponding conjugate of tau with the gold-standard carrier KLH, which is the mimic of the conjugate having successfully completed phases 1/2 clinical trials. The antibody response induced by Qβ-tau could be boosted in some mice one year after immunization, suggesting that long-term immunological memory was generated. The antibodies elicited showed a preference for tau with exposed MTBR regions mimicking aggregation-prone pathological tau. Additionally, immunostaining showed that elicited antibodies were capable of recognizing tau in brain samples of human AD patients. In addition to testing in wild-type C57BL/6 mice, the mQβ-tau vaccine was efficacious in human tau transgenic rTG4510 mice. Despite human tau being an endogenous protein in these mice, high antitau IgG titers were elicited. Furthermore, vaccination with mQβ-tau significantly reduced levels of tau protein and inflammatory cytokines in mouse brains. Vaccination enhanced the cognitive function of immunized mice, and the vaccine was immunogenic in a llama, suggesting its translational potential. Further studies are needed to establish the safety and selectivity of such a vaccine for future clinical application to the human population.

It should be pointed out that Qβ-based vaccines targeting phosphorylated tau peptides outside the MTBR region have been reported, which were found to be safe, immunogenic, and efficacious in reducing disease severity in mice and rhesus macaques.^18,19,21^ The MTBR epitope studied in this work is distinct from the reported phosphorylated tau. These results together suggest that multiple tau epitopes can be targeted using the Qβ platform. An intriguing future direction is the combination of multiple tau epitope-based vaccines to further enhance the vaccine efficacy. In addition, these vaccines may be combined with other tau-lowering strategies such as antisense oligonucleotides to mitigate symptoms and improve treatment outcomes against Alzheimer’s disease.^62^

## Materials and Methods

### General Methods

Centrifugal filter units of 10,000 and 100,000 molecular weight cutoff (MWCO) were purchased from EMD Millipore. Fast protein liquid chromatography (FPLC) was performed on a GE ÄKTA Explorer (Amersham Pharmacia) instrument equipped with a Superose-6 column. For the characterization of Qβ constructs, liquid chromatography–mass spectrometry (LCMS) analysis was performed. The samples for LCMS were prepared as follows: a mixture of 1:1 v/v of 40 μg mL^–1^ of sample stock solution and 100 mM dithiothreitol (DTT) incubated in a water bath at 37 °C for 30 min. One drop of 50% formic acid was added to the mixture. LCMS was performed on Waters Xevo G2-XS quadrupole/time-of-flight UPLC/MS/MS. The liquid chromatography was performed using an ACUITY UPLC Peptide BEH C18 column, 130 Å, 1.7 μm, 2.1 mm × 150 mm, using gradient eluent from 95% 0.1% formic acid in CH_3_CN (0.3 mL min^–1^ flow rate) at a column temperature of 40 °C. The spectra were deconvoluted using MaxEnt1. Protein concentration was measured using the Coomassie Plus Protein Reagent (Bradford Assay, Pierce) with BSA as the standard. p-Tau was prepared as previously described.^42^ The human tau MTBR peptide was purchased from Genscript. Human tissues were obtained from the NIH NeuroBioBank.

### Synthesis of Qβ-Tau and mQβ-Tau

Qβ and mQβ were expressed and purified as previously described.^38,59^ GMBS (0.2 mg, 0.71 mmol, 5 equiv per CP corresponding to 900 equiv per capsid) was dissolved in DMSO (10 μL). The solution was slowly added to a solution of Qβ/mQβ (10 mg mL^–1^ in PBS, pH = 7, 0.1 mL). The reaction was mixed on a nutating mixer in the dark for 1 h at room temperature. The Qβ-GMBS/mQβ-GMBS was washed twice by centrifuge filtration using Amicon Ultra 0.5 mL centrifuge filters (MWCO = 10 kDa). The MTBR tau peptide (0.46 mg, 0.35 mmol, 5 equiv with respect to CP) was dissolved in DMSO (10 μL). The solution was added to the solution of Qβ-GMBS/mQβ-GMBS (10 mg mL^–1^ in PBS, pH = 7, 0.1 mL). The reaction was mixed on a nutating mixer in the dark for 2 h at room temperature. The Qβ-tau/mQβ-tau was purified by centrifuge filtration using Amicron Ultra 0.5 mL centrifuge filters (MWCO = 10 kDa). Mass recovery of Qβ-tau/mQβ-tau was typically >90% as measured by Bradford assay using BSA as a standard. The average degree of functionalization was 401 antigens per Qβ (an average of 2.2 antigens per CP, Figure S1) or 244 antigens per mQβ (an average of 1.4 antigens per CP) as determined by LCMS. Increasing the equivalents of GMBS (15 equiv) and tau peptide (10 equiv) increased the loading level of mQβ-tau to 393 antigens per mQβ (an average of 2.2 antigens per CP, Figure S3).

### Synthesis of KLH-Tau

Synthesis of KLH-tau was performed as previously described.^23^ A solution of GMBS (40 mg mL^–1^ in DMSO, 50 μL) was added to a solution of KLH (10 mg mL^–1^ in PBS, pH = 7, 1 mL). The reaction was mixed on a nutating mixer in the dark for 2 h at room temperature. The KLH-GMBS was washed twice by centrifuge filtration using Amicon Ultra 0.5 mL centrifuge filters (MWCO = 10 kDa). The solution was adjusted to a final volume of 0.95 mL. A solution of the MTBR tau peptide (400 mg mL^–1^ in DMSO, 50 μL) was added to the solution, and the reaction was mixed on a nutating mixer in the dark for 2 h at room temperature. The KLH-tau was purified by centrifuge filtration using Amicon Ultra 0.5 mL centrifuge filters (MWCO = 10 kDa). Peptide loading level was determined by quantifying the recovered unreacted peptide via HPLC. The degree of functionalization was found to be 740 antigens per KLH particle.

### Synthesis of BSA-Tau

GMBS (2.1 mg, 7.6 μmol, 50 equiv with respect to BSA) was dissolved in DMSO (100 μL). The solution was slowly added to a solution of BSA (10 mg mL^–1^ in PBS, pH = 7.0, 1 mL). The reaction was mixed on a nutating mixer in the dark for 1 h at room temperature. The BSA-GMBS was washed twice by centrifuge filtration using Amicon Ultra 0.5 mL centrifuge filters (MWCO = 10 kDa). Tau peptide (5.0 mg, 3.8 μmol, 25 equiv with respect to BSA) was dissolved in DMSO (100 μL). The solution was added to the solution of BSA-GMBS (10 mg mL^–1^ in PBS, pH = 7.0, 1 mL). The reaction was mixed on a nutating mixer in the dark for 2 h at room temperature. The BSA-tau was purified by centrifuge filtration using Amicon Ultra 0.5 mL centrifuge filters (MWCO = 10 kDa). Mass recovery of BSA-tau was typically >90% as measured by the Bradford assay using BSA as a standard. The degree of functionalization was 17.5 antigens per BSA as determined by MALDI-TOF (Figure S2).

### Expression and Purification of Tau_151–391_ and 2N4R Tau

BL21 (DE3) *E. coli* transfected with a PET28b plasmid containing the gene for either tau_151–391_ with a TEV cleavable His-tag or 2N4R tau (Genscript) were grown in LB media supplemented with kanamycin (20 mg/L) to an OD_600_ of 0.7–1.0. Expression was induced by the addition of isopropyl β-D-1-thiogalactopyranoside (IPTG, 1 mM), and expression was run for 6 h at 37 °C with shaking. Cells were pelleted by centrifugation at 7000*g* for 20 min. Pelleted cells were resuspended in the lysis buffer (20 mM Tris, 100 mM NaCl, 1 mM EDTA, 1 mM PMSF, 20 mL/L of culture) and stored at −80 °C until purification.

Cells were thawed, and all subsequent steps were performed at 4 °C. Cells were sonicated at 30% power for 10 min with 10 s on and 10 s off (total = 20 min). The lysate was centrifuged at 10000*g* for 10 min. The supernatant was collected and heated in a boiling water bath for 30 min, centrifuged for 10 min at 10,000*g*, and collected. The pH value of the supernatant was adjusted to pH 8 and loaded onto a nickel column. The column was washed twice with 5 column volumes of washing buffer (20 mM Tris HCl, 500 mM NaCl, pH 8). The protein of interest was then eluted twice using 5 column volumes of elution buffer (20 mM Tris HCl, 500 mM NaCl, 100 mM imidazole, and 8 M urea). Fractions containing the protein of interest were snap-frozen in liquid nitrogen and lyophilized. Before use, samples were resuspended in PBS and washed with a 10 or 30 kDa MWCO centrifuge filter to remove the elution buffer.

### Western Blotting

Various tau constructs (tauO, monomeric tau, or p-tau, 1 μg) were run on a 4–15% SDS-PAGE gel under reducing conditions (220 V for 30 min at RT). Following gel electrophoresis, the proteins were transferred to a prewetted PVDF membrane (60 V for 90 min at 4 °C). The successful transfer was confirmed by the use of a prestained M.W. ladder. Blots were blocked overnight using blocking buffer (EveryBlot, Bio-Rad). After blocking, blots were washed 3 times with Tri*s* Buffered Saline with Tween-20 (TBST) for 15 min for each wash. Blots were stained with primary antibodies (immunized sera or Tau5) diluted (1:500 or 1:1000, respectively) in the blocking buffer for 2 h at RT. Unbound primary antibodies were removed by washing 3 times with TBST. Primary antibodies were detected by staining with horseradish peroxidase (HRP)-conjugated goat antimouse IgG diluted (1:1000) in the blocking buffer for 1 h and washed 3 times with TBST for 15 min each wash. Staining was detected using ECL spray (Prometheus Protein Biology, catalog # 20-300S) and imaged on a ChemiDoc imaging system (Bio-Rad).

### Immunization of Mice

Female mice (C57BL/6 or rTg4510) aged 6–10 weeks were used for studies. All animal experiments were performed in accordance with the guidelines of the Institutional Animal Care and Use Committee (IACUC) of Michigan State University. The animal usage protocol approval number is PROTO201900423. Female rTg4510 mice were used as it is known that this strain of female mice is associated with more severe diseases and AD-like symptoms as compared to the corresponding male mice.^60^

In all studies, mice were subcutaneously injected under the scruff on day 0 with 0.1 mL of various vaccine constructs in PBS containing Alum (Imject Alum, Thermo Scientific, catalog #: 77161, 1 mg) (50 μL) for each mouse. Vaccine doses were prepared by vortexing the mixture at 4 °C until an emulsion formed. Boosters were given subcutaneously with the same amounts of vaccines and Alum under the scruff on days 14 and 28. All vaccine doses had the same amount of antigen (9.6 nmol). Serum samples were collected on days 0 (before immunization), 21, 35, 70, and 90. C57BL/6 mice received an additional boost on day 365, along with additional serum samples being collected on days 365 (before the last boost) and 379.

### Immunohistochemistry

Specimens were processed, embedded in paraffin, and sectioned on a rotary microtome at 4 μm. Sections were placed on charged slides and dried at 56 °C overnight. The slides were subsequently deparaffinized in xylene and hydrated through descending grades of ethyl alcohol to distilled water. Slides were placed in Tris-buffered saline (TBS), pH 7.4 (Scytek Laboratories, Logan, UT) for 5 min for pH adjustment. Following TBS, heat-induced epitope retrieval in citrate plus, pH 6.0 (Scytek) was performed in a vegetable steamer at 100 °C for 30 min, which was followed by incubation for 10 min at room temperature and several rinses with distilled water. Endogenous peroxidase was blocked utilizing 3% hydrogen peroxide/methanol bath for 30 min followed by running tap and distilled water rinses. Following pretreatments, standard micropolymer complex staining steps were performed at room temperature on the IntelliPath Flex Autostainer. All staining steps were followed by rinses in TBS Autowash buffer (Biocare Medical, Concord, CA). After blocking for nonspecific protein with IP FLX Background Punisher (Biocare) for 5 min; sections were incubated with specific primary (1:300 Tau5, 1:1000 D35 sera, or 1:1000 preimmune sera) in normal antibody diluent (NAD-Scytek). MACH3Mouse Probe and Polymer (Biocare) were then added and incubated for 10 min each, followed by reaction development with Romulin AEC (Biocare) for 5 min and counterstained with Cat Hematoxylin for 5 min.

### Immunofluorescence

Tissue sections were fixed in chilled methanol for 10 min and washed 3 times in PBS supplemented with 0.25% Triton X-100 (0.25% PBST) for 5 min. Following washing, the sections were washed in 70% ethanol (EtOH). Autofluorescence was blocked by treating tissue sections with TrueBlack (Biotium, catalog no. 23007) for 5 min and then washed with 70% EtOH three times for 1 min and once with 0.25% PBST for 10 min. Tissue sections were blocked with 5% goat serum in incubation buffer (0.25% PBST supplemented with 5% BSA) for 1 h in a humidity chamber and washed 3 times in 0.25% PBST for 5 min. Tissue sections were incubated with primary antibodies diluted in incubation buffer overnight at 4 °C in a humidity chamber and washed 3 times in 0.25% PBST for 5 min. The positive control rabbit total tau antibody was purchased from Sigma-Aldrich (cat # ABN454). Primary antibodies were stained using goat antimouse IgG Alexa Fluor 488 and goat antirabbit IgG Alexa Fluor 568 diluted in incubation buffer for 1 h at RT in a humidity chamber and washed 3 times with 0.25% PBST for 10 min. Nuclei were stained by incubation with DAPI in incubation buffer for 5 min at RT in a humidity chamber and washed 3 times with 0.25% PBST for 5 min. Coverslips were mounted using Fluoromount G and sealed with nail polish.

### Llama Immunization

All work was completed under contract by ProSci Inc. A female llama (8 years old) was immunized subcutaneously on day 0 with 200 μg of mQβ-tau (corresponding to 40.6 μg or 31.0 nmol of the peptide) and Complete Freund’s Adjuvant. On days 21, 49, and 77, the llama was boosted subcutaneously with 100 μg of mQβ-tau and Incomplete Freund’s Adjuvant. On days 0, 28, 56, and 84, blood was collected.

### Evaluation of Antibody Titers by ELISA

The Nunc MaxiSorp flat-bottom 96-well microtiter plates were coated with 10 μg mL^–1^ of BSA-tau, 2N4R tau, tau_151–394_, or p-tau (100 μL/well) in a NaHCO_3_/Na_2_CO_3_ buffer (0.05 M, pH 9.6) by incubation at 4 °C overnight. The coated plates were washed with PBS/0.5% Tween-20 (PBST) (4 × 200 μL) and blocked with 1% BSA in PBS (100 μL/well) at RT for 1 h. The plates were washed again with PBST (4 × 200 μL) and incubated with serial dilutions of mouse sera in 0.1% BSA/PBS (100 μL/well, 4 wells for each dilution). The plates were incubated for 2 h at 37 °C and then washed with PBST (4 × 200 μL). A 1:2000 dilution of HRP-conjugated goat antimouse IgG, IgG1, IgG2b, IgG2c, IgG3, or IgM (Jackson ImmunoResearch Laboratory) or HRP-conjugated goat antialpaca IgG1 or IgG2/3 (Jackson ImmunoResearch Laboratory) in 0.1% BSA/PBS (100 μL) was added to the wells, respectively, to determine the titers of antibodies generated. The plates were incubated for 1 h at 37 °C and then washed with PBST (4 × 200 μL). A solution of enzymatic substrate 3,3′,5,5′-tetramethylbenzidine (TMB, 200 μL) was added to the plates (for one plate: 5 mg of TMB was dissolved in 2 mL of DMSO plus 18 mL of citric acid buffer containing 20 μL of H_2_O_2_). Color was allowed to develop for 15 min and then quenched by adding 50 μL of 0.5 M H_2_SO_4_. The readout was measured at 450 nm using a microplate reader. The titer number was calculated as the highest fold of dilution giving the optical absorbance value equal to the average of the blank well plus three times the standard deviation.

### Isolation of Soluble Tau and Sarkosyl-Insoluble Tau

Dorsal hippocampal subregion CA1 brain tissue was homogenized in 10-fold weight excess of ice-cold extraction buffer (20 mM Tris, pH 7.4, 800 mM NaCl, 1 mM ethylene glycol tetraacetic acid), 1 mM ethylenediaminetetraacetic acid 0.5% β-mercaptoethanol, 10% sucrose, 1 mM Na_3_VO_4_, and 20 mM NaF, supplemented with a cOmplete Protease Inhibitor Cocktail Tablet (Roche Diagnostics). After being incubated on ice for 5 min, the homogenates were cleared by centrifugation at 20,000*g* for 20 min at 4 °C. The supernatants were collected, and the total protein concentration was determined using a Bio-Rad protein assay (Bio-Rad Laboratories, Hercules, CA). This supernatant (designed 1S) contained a soluble tau fraction. Subsequently, solid sarkosyl (N-lauroylsarcosine sodium salt; Sigma-Aldrich) was added to the 1S supernatant to achieve 1% concentration and then stirred for 1 h. Thereafter, it was centrifuged at 100,000*g* for 1.5 h at RT. Following centrifugation, pellets were gently rinsed with 1 mL of the extraction buffer and centrifuged at 100,000*g* for 20 min at RT. The pellets containing sarkosyl-insoluble tau fractions were dissolved in SDS-PAGE loading buffer to a final volume corresponding to the 1/50 volume of the 1S supernatant.

### Real-Time Quantitative PCR

Mice (*N* = 10/treatment group, rTg4510, age 15 weeks) were euthanized with CO_2_, followed by rapid decapitation. Whole brains were flash frozen and stored at −80 °C. Brains were then sectioned at 200 μm via cryostat and thaw-mounted onto slides. The dorsal hippocampal subregion CA1 was punched from the slices using a 1 mm micropuncher (Harris Micropunch, Hatfield, PA). RNA was extracted with Qiagen RNeasy Plus Mini kits (Qiagen, Valencia, CA, Cat# 74134) and quantified using a Qubit Flex Fluorometer (Thermo Fisher Scientific). The RNAs were converted to cDNA using a high-capacity cDNA Reverse Transcription Kit (Applied Biosystems, CA, Cat# 4368814).

The RT-qPCR was conducted using a SYBR green Master mix consisting of 5 ng cDNA and 0.25 μM of each primer set. Reactions were performed in triplicate. Forward and reverse primer sequences used were 5′-AAA CCA CAG TTC CGC AGA GA-3′ and 5′-CGT GTT CAC CAG CTG GCT TA-3′, respectively, for CD11b; 5′-CCA CGG CCT TCC CTA CTT C-3′ and 5′-TTG GGA GTG GTA TCC TCT GTG A-3′, respectively, for IL-6; 5′-TTC GGC TAC CCC AAG TTC AT −3′ and 5′-CGC ACG TAG TTC CGC TTT C-3′, respectively, for TNF-α; 5′-CAG TCC CAG CGT CGT GAT TA-3′ and 5′-TGG CCT CCC ATC TCC TTC AT-3′, respectively, for *Hprt-1*. CT values were automated by the QuantStudio-5 Real-Time PCR System analysis software (Applied Biosystems, 272511214). ΔCT was used to calculate the relative expression of the targeted gene over the housekeeping gene (HPRT). ΔΔCT was then used to calculate the relative gene expression levels in the PBS, mQβ/tau admixture, and mQβ-tau vaccine group, respectively.

### Immunoblot Analysis of Soluble Tau and Sarkosyl-Insoluble Tau

Samples of sarkosyl-insoluble tau fractions were dissolved in 1× SDS sample loading buffer in a 1/50 volume of the soluble fraction and heated at 95 °C for 5 min. Each sample (6 μL) was then loaded onto 5–20% gradient SDS polyacrylamide gels and electrophoresed in a Tris-glycine SDS buffer system for 30 min at 200 V. Proteins were transferred to PVDF membrane (1 h at 60 V in 10 mM N-cyclohexyl-3-aminopropanesulfonic acid, pH 12), and, after blocking in 10% fat-free dry milk in PBS for 2 h at room temperature, the membrane was incubated for 1 h with mAb (Tau5) obtained from abcam, as previously described for quantification of tau protein in brain tissues.^61^ After washes, HRP-conjugated goat antimouse IgG (Cat # 115-035-003 Jackson ImmunoResearch) diluted 1:2000 in PBS was used as a secondary antibody. Incubation (1 h at room temperature) was followed by washing with TBST. The blots were developed with WesternBright ECL (advansta), and the signal was detected using the BIO-RAD ChemiDoc MP imaging System. The signal intensities were quantified using AIDA software and then statistically evaluated using an unpaired *t-*test.

### Novel Object Recognition Test

rTg4510 mice were fully immunized with mQβ-tau (Scheme S1). On day 42 after the prime immunization, a novel object recognition test was performed, which took 4 days. On days 1–3 of this 4-day protocol, animals were acclimated to an open 70 cm^2^ arena with two identical objects (rectangle blocks) in opposite corners of the arena for 5 min. Twenty-four hours after day 3, the mice were exposed to two identical objects (rectangle blocks) for 5 min in opposite corners of the arena. After a 45 min intermission, the mice were exposed to the same arena with a novel object (pencil topper eraser) replacing 1 of the original objects. The testing session was video recorded with the time each mouse spent exploring the novel or familiar object scored by a researcher blind to the treatment conditions. The percentage of time the mouse spent with the novel object was calculated (as the discrimination index). Results presented were acquired with Ethovision XT8 (Noldus, Netherlands) and then processed in Excel and GraphPad Prism 6.

## Supplementary Material



## Notes

Characterization data for vaccine constructs, anticarrier titers, titer curves from llama immunization, tau purification analysis, and representative Western blots from brain homogenate analysis (PDF)

## ◆

School of Natural Sciences, Northwest Missouri State University, Maryville, MO 64468, USA

## References

[1] Alzheimer’s Association . Alzheimer’s Disease Facts and Figures 2025 https://www.alz.org/getmedia/ef8f48f9-ad36-48ea-87f9-b74034635c1e/alzheimers-facts-and-figures.pdf (accessed Nov 23, 2025).

[2] Moloney C.
M., Lowe V. J., Murray M. E. (2021). Visualization of Neurofibrillary
Tangle Maturity in Alzheimer’s Disease: a Clinicopathologic
Perspective for Biomarker Research. Alzheimer’s
Dementia.

[3] Mukrasch M.
D., Bibow S., Korukottu J., Jeganathan S., Biernat J., Griesinger C., Mandelkow E., Zweckstetter M. (2009). Structural Polymorphism of 441-Residue
Tau at Single
Residue Resolution. PLOS Biol..

[4] Jeganathan S., von Bergen M., Brutlach H., Steinhoff H.-J., Mandelkow E. (2006). Global Hairpin
Folding of Tau in Solution. Biochemistry.

[5] Gong C. X., Iqbal K. (2008). Hyperphosphorylation
of microtubule-associated protein tau: a promising
therapeutic target for Alzheimer disease. Curr.
Med. Chem..

[6] Noble W., Hanger D. P., Miller C. C., Lovestone S. (2013). The Importance
of Tau Phosphorylation for Neurodegenerative Diseases. Front. Neurol..

[7] Fischer D., Mukrasch M. D., Biernat J., Bibow S., Blackledge M., Griesinger C., Mandelkow E., Zweckstetter M. (2009). Conformational
Changes Specific for Pseudophosphorylation at Serine 262 Selectively
Impair Binding of Tau to Microtubules. Biochemistry.

[8] Haase C., Stieler J. T., Arendt T., Holzer M. (2004). Pseudophosphorylation
of tau protein alters its ability for self-aggregation. J. Neurochem..

[9] Cowan C. M., Mudher A. (2013). Are Tau Aggregates
Toxic or Protective in Tauopathies?. Front.
Neurol..

[10] Shafiei S. S., Guerrero-Muñoz M. J., Castillo-Carranza D. L. (2017). Tau Oligomers:
Cytotoxicity, Propagation, and Mitochondrial Damage. Front. Aging Neurosci..

[11] Lasagna-Reeves C. A., Castillo-Carranza D. L., Sengupta U., Clos A. L., Jackson G. R., Keyed R. (2011). Tau Oligomers
Impair Memory and Induce Synaptic and Mitochondrial
Dysfunction in Wild-Type Mice. Mol. Neurodegener..

[12] Kontsekova E., Zilka N., Kovacech B., Skrabana R., Novak M. (2014). Identification
of Structural Determinants on Tau Protein Essential for its Pathological
Function: Novel Therapeutic Target for Tau Immunotherapy in Alzheimer’s
Disease. Alzheimer’s Res. Ther..

[13] Yanamandra K., Jiang H., Mahan T. E., Maloney S. E., Wozniak D. F., Diamond M. I., Holtzman D. M. (2015). Anti-tau antibody reduces insoluble
tau and decreases brain atrophy. Ann. Clin.
Transl. Neurol..

[14] Gaikwad S., Puangmalai N., Sonawane M., Montalbano M., Price R., Iyer M. S., Ray A., Moreno S., Kayed R. (2024). Nasal tau immunotherapy clears intracellular tau pathology and improves
cognitive functions in aged tauopathy mice. Sci. Transl. Med..

[15] Wu R., Sun F., Zhang W., Ren J., Liu G.-H. (2024). Targeting aging
and age-related diseases with vaccines. Nat.
Aging.

[16] Nicholas C. C., Petr N., Duygu T., Branislav K., Jozef H., Eva K., Michal F., Stefan R., Howard H. F., Reinhold S., Bengt W., Norbert Z. (2024). Efficacy assessment
of an active tau immunotherapy in Alzheimer’s disease patients
with amyloid and tau pathology: a post hoc analysis of the “ADAMANT”
randomised, placebo-controlled, double-blind, multi-centre, phase
2 clinical trial. eBioMedicine.

[17] Novak P., Kovacech B., Katina S., Schmidt R., Scheltens P., Kontsekova E., Ropele S., Fialova L., Kramberger M., Paulenka-Ivanovova N., Smisek M., Hanes J., Stevens E., Kovac A., Sutovsky S., Parrak V., Koson P., Prcina M., Galba J., Cente M., Hromadka T., Filipcik P., Piestansky J., Samcova M., Prenn-Gologranc C., Sivak R., Froelich L., Fresser M., Rakusa M., Harrison J., Hort J., Otto M., Tosun D., Ondrus M., Winblad B., Novak M., Zilka N. (2021). ADAMANT: A
Placebo-Controlled Randomized Phase 2 study of AADvac1, an Active
Immunotherapy Against Pathological Tau in Alzheimer’s Disease. Nat. Aging.

[18] Maphis N. M., Hulse J., Peabody J., Dadras S., Whelpley M. J., Kandath M., Wilson C., Hobson S., Thompson J., Poolsup S., Beckman D., Ott S. P., Watanabe J. W., Usachenko J. L., Van Rompay K. K., Morrison J., Selwyn R., Rosenberg G., Knoefel J., Chackerian B., Bhaskar K. (2025). Targeting of phosphorylated
tau at threonine 181 by
a Qβ virus-like particle vaccine is safe, highly immunogenic,
and reduces disease severity in mice and rhesus macaques. Alzheimer’s Dementia.

[19] Hulse J. P., Maphis N. M., Peabody J., Bondu V., Chackerian B., Bhaskar K. (2025). pS396/pS404 (PHF1) tau vaccine outperforms pS199/pS202
(AT8) in rTg4510 tauopathy model. npj Vaccines.

[20] Theunis C., Crespo-Biel N., Gafner V., Pihlgren M., López-Deber M. P., Reis P., Hickman D. T., Adolfsson O., Chuard N., Ndao D. M., Borghgraef P., Devijver H., Van Leuven F., Pfeifer A., Muhs A. (2013). Efficacy and
Safety of A Liposome-Based Vaccine against Protein Tau, Assessed in
Tau.P301L Mice That Model Tauopathy. PLoS One.

[21] Maphis N. M., Peabody J., Crossey E., Jiang S., Jamaleddin
Ahmad F. A., Alvarez M., Mansoor S. K., Yaney A., Yang Y., Sillerud L. O., Wilson C. M., Selwyn R., Brigman J. L., Cannon J. L., Peabody D. S., Chackerian B., Bhaskar K. (2019). Qß Virus-like particle-based vaccine induces robust
immunity and protects against tauopathy. NPJ
Vaccines.

[22] Davtyan H., Chen W. W., Zagorski K., Davis J., Petrushina I., Kazarian K., Cribbs D. H., Agadjanyan M. G., Blurton-Jones M., Ghochikyan A. (2017). MultiTEP platform-based
DNA epitope
vaccine targeting N-terminus of tau induces strong immune responses
and reduces tau pathology in THY-Tau22 mice. Vaccine.

[23] Kontsekova E., Zilka N., Kovacech B., Novak P., Novak M. (2014). First-in-Man
Tau Vaccine Targeting Structural Determinants Essential for Pathological
Tau-Tau Interaction Reduces Tau Oligomerization and Neurofibrillary
Degeneration in an Alzheimer’s Disease Model. Alzheimer’s Res. Ther..

[24] Novak P., Schmidt R., Kontsekova E., Zilka N., Kovacech B., Skrabana R., Vince-Kazmerova Z., Stanislav K., Fialova L., Prcina M., Parrak V., Dal-Bianco P., Brunner M., Staffen W., Rainer M., Ondrus M., Ropele S., Smisek M., Sivak R., Winblad B., Novak M. (2017). Safety and Immunogenicity of the
Tau Vaccine AADvac1 in Patients
with Alzheimer’s Disease: A Randomized, Double-Blind, Placebo-Controlled,
Phase 1 Trial. Lancet Neurol..

[25] Novak P., Schmidt R., Kontsekova E., Kovacech B., Smolek T., Katina S., Fialova L., Prcina M., Parrak V., Dal-Bianco P., Brunner M., Staffen W., Rainer M., Ondrus M., Ropele S., Smisek M., Sivak R., Zilka N., Winblad B., Novak M. (2018). FUNDAMANT: An Interventional
72-Week Phase 1 Follow-Up Study of AADvac1, an Active Immunotherapy
Against Tau Protein Pathology in Alzheimer’s Disease. Alzheimer’s Res. Ther..

[26] McFall-Boegeman H., Huang X. (2022). Mechanisms of cellular and humoral immunity through the lens of VLP-based
vaccines. Expert Rev. Vaccines.

[27] Cornuz J., Zwahlen S., Jungi W. F., Osterwalder J., Klingler K., van Melle G., Bangala Y., Guessous I., Müller P., Willers J., Maurer P., Bachmann M. F., Cerny T. (2008). A Vaccine
against Nicotine for Smoking Cessation: A Randomized Controlled
Trial. PLoS One.

[28] Polonskaya Z., Deng S., Sarkar A., Kain L., Comellas-Aragones M., McKay C. S., Kaczanowska K., Holt M., McBride R., Palomo V., Self K. M., Taylor S., Irimia A., Mehta S. R., Dan J. M., Brigger M., Crotty S., Schoenberger S. P., Paulson J. C., Wilson I. A., Savage P. B., Finn M. G., Teyton L. (2017). T cells control the generation of
nanomolar-affinity anti-glycan antibodies. J.
Clin. Invest..

[29] Tan Z., Yang C., Lin P.-h., Ramadan S., Yang W., Rashidi Z., Lang S., Shafieichaharberoud F., Gao J., Pan X., Soloff N., Wu X., Bolin S., Pyeon D., Huang X. (2024). Inducing Long Lasting B Cell and
T Cell Immunity Against Multiple Variants of SARS-CoV-2 Through Mutant
Bacteriophage Qβ—Receptor Binding Domain Conjugate. Adv. Healthcare Mater..

[30] Bachmann M. F., Jennings G. T. (2010). Vaccine delivery:
a matter of size, geometry, kinetics
and molecular patterns. Nat. Rev. Immunol..

[31] Shafieichaharberoud F., Lang S., Whalen C., Rivera Quiles C., Purcell L., Talbot C., Wang P., Norton E. B., Mazei-Robison M., Sulima A., Jacobson A. E., Rice K. C., Matyas G. R., Huang X. (2024). Enhancing Protective
Antibodies against
Opioids through Antigen Display on Virus-like Particles. Bioconjugate Chem..

[32] Tan Z., Yang W., O’Brien N. A., Pan X., Ramadan S., Marsh T., Hammer N., Cywes-Bentley C., Vinacur M., Pier G. B., Gildersleeve J. C., Huang X. (2024). A comprehensive synthetic library of poly-N-acetyl glucosamines enabled
vaccine against lethal challenges of Staphylococcus aureus. Nat. Commun..

[33] Lim F., Spingola M., Peabody D. S. (1996). The RNA-binding
Site of Bacteriophage
Qβ Coat Protein. J. Biol. Chem..

[34] Golmohammadi R., Fridborg K., Bundule M., Valegård K., Liljas L. (1996). The Crystal Structure of Bacteriophage
Qβ at
3.5 Å Resolution. Structure..

[35] Braun M., Jandus C., Maurer P., Hammann-Haenni A., Schwarz K., Bachmann M. F., Speiser D. E., Romero P. (2012). Virus-Like
Particles Induce Robust Human T-Helper Cell Responses. Eur. J. Immunol..

[36] Wang P., Huo C.-x., Lang S., Caution K., Nick S. T., Dubey P., Deora R., Huang X. (2020). Chemical Synthesis
and Immunological Evaluation of a Pentasaccharide Bearing Multiple
Rare Sugars as a Potential Anti-pertussis Vaccine. Angew. Chem., Int. Ed..

[37] Wu X., Ye J., DeLaitsch A. T., Rashidijahanabad Z., Lang S., Kakeshpour T., Zhao Y., Ramadan S., Saavedra P. V., Yuzbasiyan-Gurkan V., Kavunja H., Cao H., Gildersleeve J. C., Huang X. (2021). Chemoenzymatic Synthesis of 9NHAc-GD2 Antigen to Overcome the Hydrolytic
Instability of O-Acetylated-GD2 for Anticancer Conjugate Vaccine Development. Angew. Chem., Int. Ed..

[38] Sungsuwan S., Wu X., Shaw V., Kavunja H., McFall-Boegeman H., Rashidijahanabad Z., Tan Z., Lang S., Nick S. T., Lin P.-H., Yin Z., Ramadan S., Jin X., Huang X. (2022). Structure Guided Design of the Bacteriophage Qβ Mutants as
Next Generation Carriers for Conjugate Vaccines. ACS. Chem. Biol..

[39] Lasagna-Reeves C. A., Castillo-Carranza D. L., Sengupta U., Guerrero-Munoz M. J., Kiritoshi T., Neugebauer V., Jackson G. R., Kayed R. (2012). Alzheimer
brain-derived tau oligomers propagate pathology from endogenous tau. Sci. Rep..

[40] Gu J., Xu W., Jin N., Li L., Zhou Y., Chu D., Gong C.-X., Iqbal K., Liu F. (2020). Truncation of Tau selectively
facilitates its pathological activities. J.
Biol. Chem..

[41] Filipcik P., Zilka N., Bugos O., Kucerak J., Koson P., Novak P., Novak M. (2012). First transgenic rat model developing
progressive cortical neurofibrillary tangles. Neurobiol. Aging..

[42] Liu M., Sui D., Dexheimer T., Hovde S., Deng X., Wang K.-W., Lin H. L., Chien H.-T., Kweon H. K., Kuo N. S., Ayoub C. A., Jimenez-Harrison D., Andrews P. C., Kwok R., Bochar D. A., Kuret J., Fortin J., Tsay Y.-G., Kuo M.-H. (2020). Hyperphosphorylation
Renders Tau Prone to Aggregate
and to Cause Cell Death. Mol. Neurobiol..

[43] Porzig R., Singer D., Hoffmann R. (2007). Epitope mapping
of mAbs AT8 and Tau5
directed against hyperphosphorylated regions of the human tau protein. Biochem. Biophys. Res. Commun..

[44] Schutze M. P., Deriaud E., Przewlocki G., LeClerc C. (1989). Carrier-induced epitopic
suppression is initiated through clonal dominance. J. Immunol..

[45] Jegerlehner A., Wiesel M., Dietmeier K., Zabel F., Gatto D., Saudan P., Bachmann M. F. (2010). Carrier
induced epitopic suppression
of antibody responses induced by virus-like particles is a dynamic
phenomenon caused by carrier-specific antibodies. Vaccine..

[46] McCluskie M. J., Evans D. M., Zhang N., Benoit M., McElhiney S. P., Unnithan M., DeMarco S. C., Clay B., Huber C., Deora A., Thorn J. M., Stead D. R., Merson J. R., Davis H. L. (2016). The effect of preexisting anti-carrier immunity on
subsequent responses to CRM197 or Qb-VLP conjugate vaccines. Immunopharmacol. Immunotoxicol..

[47] Alves R. L., Gonçalves A., Voytyuk I., Harrison D. C. (2025). Behaviour profile
characterization of PS19 and rTg4510 tauopathy mouse models: A systematic
review and a meta-analysis. Exp. Neurol..

[48] Ramsden M., Kotilinek L., Forster C., Paulson J., McGowan E., SantaCruz K., Guimaraes A., Yue M., Lewis J., Carlson G., Hutton M., Ashe K. H. (2005). Age-Dependent Neurofibrillary
Tangle Formation, Neuron Lass, and Memory Impairment in a Mouse Model
of Human Tauopathy (P301L). J. Neurosci..

[49] Yue M., Hanna A., Wilson J., Roder H., Janus C. (2011). Sex Difference
in Pathology and Memory Decline in rTg4510 Mouse Model of Tauopathy. Neurobiol. Aging..

[50] Lueptow L. M. (2017). Novel Object
Recognition Test for the Investigation of Learning and Memory in Mice. J. Vis. Exp..

[51] Andersson C. R., Falsig J., Stavenhagen J. B., Christensen S., Kartberg F., Rosenqvist N., Finsen B., Pedersen J. T. (2019). Antibody-mediated
clearance of tau in primary mouse microglial cultures requires Fcγ-receptor
binding and functional lysosomes. Sci. Rep..

[52] Sankaranarayanan S., Barten D. M., Vana L., Devidze N., Yang L., Cadelina G., Hoque N., DeCarr L., Keenan S., Lin A., Cao Y., Snyder B., Zhang B., Nitla M., Hirschfeld G., Barrezueta N., Polson C., Wes P., Rangan V. S., Cacace A., Albright C. F., Meredith J., Trojanowski J. Q., Lee V. M. Y., Brunden K. R., Ahlijanian M. (2015). Passive Immunization with Phospho-Tau Antibodies Reduces
Tau Pathology and Functional Deficits in Two Distinct Mouse Tauopathy
Models. PLoS One.

[53] Joly-Amado A., Davtyan H., Serraneau K., Jules P., Zitnyar A., Pressman E., Zagorski K., Antonyan T., Hovakimyan A., Paek H. J., Gordon M. N., Cribbs D. H., Petrovsky N., Agadjanyan M. G., Ghochikyan A., Morgan D. (2020). Active immunization
with tau epitope in a mouse model of tauopathy induced strong antibody
response together with improvement in short memory and pSer396-tau
pathology. Neurobiol. Dis..

[54] Binder L. I., Guillozet-Bongaarts A. L., Garcia-Sierra F., Berry R. W. (2005). Tau, tangles, and Alzheimer’s
disease. Biochim. Biophys. Acta.

[55] Heneka M. T., van der Flier W. M., Jessen F., Hoozemanns J., Thal D. R., Boche D., Brosseron F., Teunissen C., Zetterberg H., Jacobs A. H., Edison P., Ramirez A., Cruchaga C., Lambert J.-C., Laza A. R., Sanchez-Mut J. V., Fischer A., Castro-Gomez S., Stein T. D., Kleineidam L., Wagner M., Neher J. J., Cunningham C., Singhrao S. K., Prinz M., Glass C. K., Schlachetzki J. C. M., Butovsky O., Kleemann K., De Jaeger P. L., Scheiblich H., Brown G. C., Landreth G., Moutinho M., Grutzendler J., Gomez-Nicola D., McManus R. M., Andreasson K., Ising C., Karabag D., Baker D. J., Liddelow S. A., Verkhratsky A., Tansey M., Monsonego A., Aigner L., Dorothée G., Nave K.-A., Simons M., Constantin G., Rosenzweig N., Pascual A., Petzold G. C., Kipnis J., Venegas C., Colonna M., Walter J., Tenner A. J., O’Banion M. K., Steinert J. R., Feinstein D. L., Sastre M., Bhaskar K., Hong S., Schafer D. P., Golde T., Ransohoff R. M., Morgan D., Breitner J., Mancuso R., Riechers S.-P. (2025). Neuroinflammation in Alzheimer disease. Nat. Rev. Immunol..

[56] Heneka M. T., Carson M. J., Khoury J. E., Landreth G. E., Brosseron F., Feinstein D. L., Jacobs A. H., Wyss-Coray T., Vitorica J., Ransohoff R. M., Herrup K., Frautschy S. A., Finsen B., Brown G. C., Verkhratsky A., Yamanaka K., Koistinaho J., Latz E., Halle A., Petzold G. C., Town T., Morgan D., Shinohara M. L., Perry V. H., Holmes C., Bazan N. G., Brooks D. J., Hunot S., Joseph B., Deigendesch N., Garaschuk O., Boddeke E., Dinarello C. A., Breitner J. C., Cole G. M., Golenbock D. T., Kummer M. P. (2015). Neuroinflammation in Alzheimer’s disease. Lancet Neurol..

[57] Muyldermans S. (2001). Single domain
camel antibodies: current status. Rev. Mol.
Biotechnol..

[58] Henry K. A., MacKenzie C. R. (2018). Antigen Recognition by Single-Domain Antibodies: Structural
Latitudes and Constraints. MABS..

[59] Fiedler J. D., Brown S. D., Lau J. L., Finn M. G. (2010). RNA-Directed Packaging
of Enzymes Within Virus-Like Particles. Angew.
Chem., Int. Ed..

[60] Yue M., Hanna A., Wilson J., Roder H., Janus C. (2011). Sex difference
in pathology and memory decline in rTg4510 mouse model of tauopathy. Neurobiol. Aging..

[61] Ye J., Yin Y., Liu H., Fang L., Tao X., Wei L., Zuo Y., Yin Y., Ke D., Wang J. Z. (2020). Tau inhibits PKA
by nuclear proteasome-dependent PKAR2α elevation with suppressed
CREB/GluA1 phosphorylation. Aging Cell..

[62] Mummery C. J., Börjesson-Hanson A., Blackburn D. J., Vijverberg E. G. B., De Deyn P. P., Ducharme S., Jonsson M., Schneider A., Rinne J. O., Ludolph A. C., Bodenschatz R., Kordasiewicz H., Swayze E. E., Fitzsimmons B., Mignon L., Moore K. M., Yun C., Baumann T., Li D., Norris D. A., Crean R., Graham D. L., Huang E., Ratti E., Bennett C. F., Junge C., Lane R. M. (2023). Tau-targeting
antisense oligonucleotide MAPTRx in mild Alzheimer’s disease:
a phase 1b, randomized, placebo-controlled trial. Nat. Med..

