# Supplementary material for: A Virus-Like-Particle-Based Conjugate Vaccine Targeting the Microtubule Binding Region of Tau Protein
Source: ACS Omega. 2026 Jul 15;11(29):43242–54. doi: 10.1021/acsomega.6c00852 (PMC13425505; doi:10.1021/acsomega.6c00852)
Supplement: Supplementary file 1 [file ao6c00852_si_001.pdf]

Supporting Information

A Virus-like-particle Based Conjugate Vaccine Targeting the Microtubule Binding Region of Tau Protein

Hunter McFall-Boegeman[a,b,#,1], Cameron Talbot[a,b,#], Mauro Montalbano,[c,d] Nicha Puangmalai,[c,d] Kuang-Wei Wang,[e] Meena Kannan,[f] Jiaming Shi,[f] and Katrina Linning-Duffy,[f] Setare Nick,[a,b] Lily Yan,[f] Min-Hao Kuo,[e] Rakez Kayed,[c,d] Xuefei Huang[a,b,g]*

[a]Department of Chemistry, Michigan State University, East Lansing, MI, 48824 USA

[b]Institute for Quantitative Health Science and Engineering, Michigan State University, East Lansing, MI, 48824 USA

[c]Mitchell Center for Neurodegenerative Diseases, University of Texas Medical Branch, Galveston, Texas, 77555 USA

[d]Departments of Neurology, Neuroscience and Cell Biology, University of Texas Medical Branch, Galveston, Texas, 77555 USA

[e]Department of Biochemistry and Molecular Biology, Michigan State University, East Lansing, MI, 48824 USA

[f]Department of Psychology, Michigan State University, East Lansing, MI, 48824 USA

[g]Department of Biomedical Engineering, Michigan State University, East Lansing, MI, 48824 USA

*Email: huangxu2@msu.edu

#Equal contribution

[1] Current address: School of Natural Sciences, Northwest Missouri State University, Maryville, MO 64468 USA

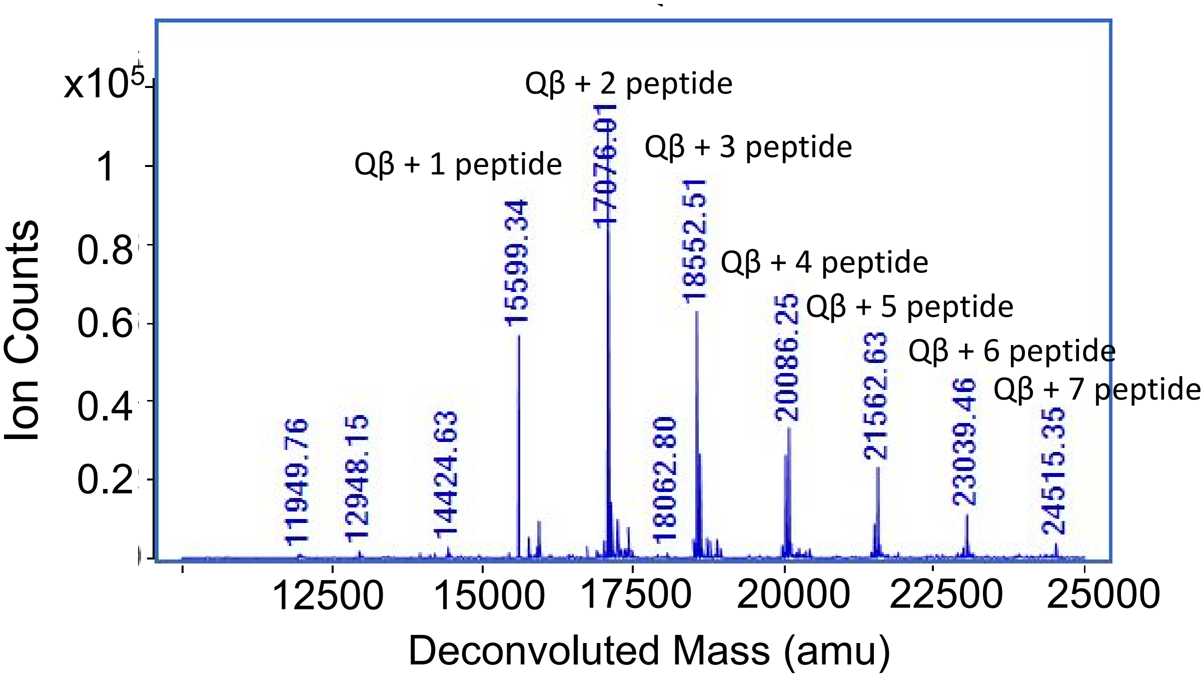

Figure S1. Deconvoluted mass spectrum from Qβ-tau. Based on the intensities of the peaks of Qβ + peptide, the average loading of antigens per capsid was calculated to be 2.2 antigens per coat protein (CP) corresponding to 401 antigens per capsid with 180 copies of CP in each capsid.

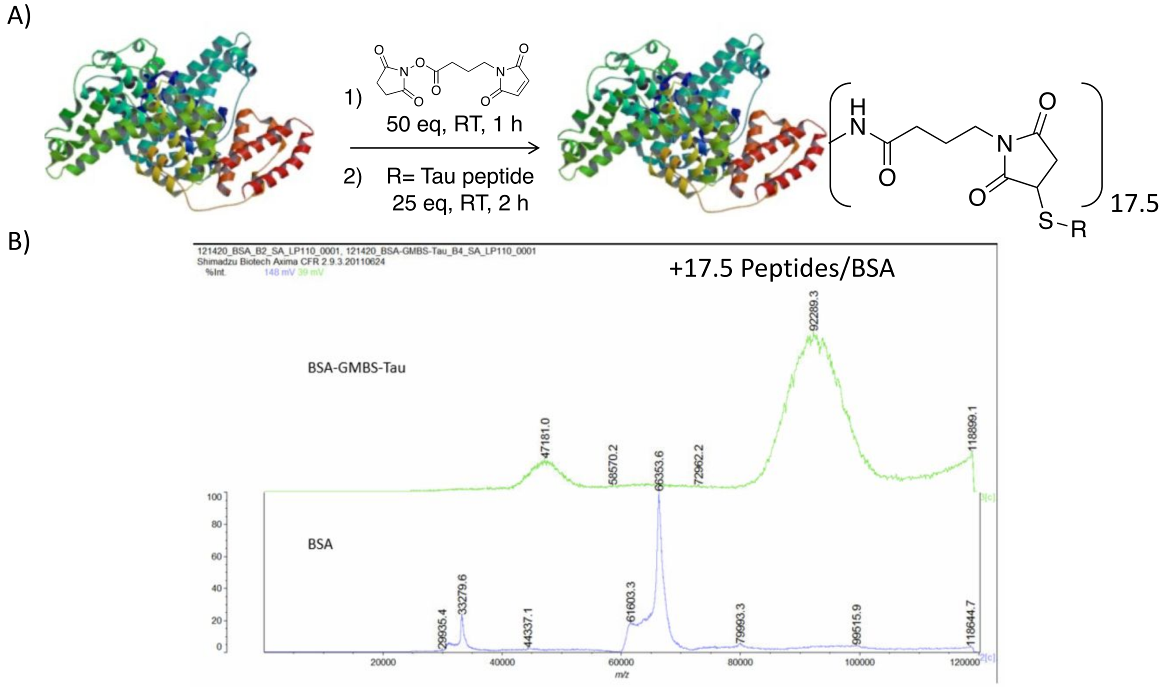

Figure S2. (A) Reaction scheme for the synthesis of BSA-tau. (B) MALDI TOF MS spectra showing BSA-tau and unmodified BSA. The peak shift corresponds to an average loading of 17.5 antigens per BSA.

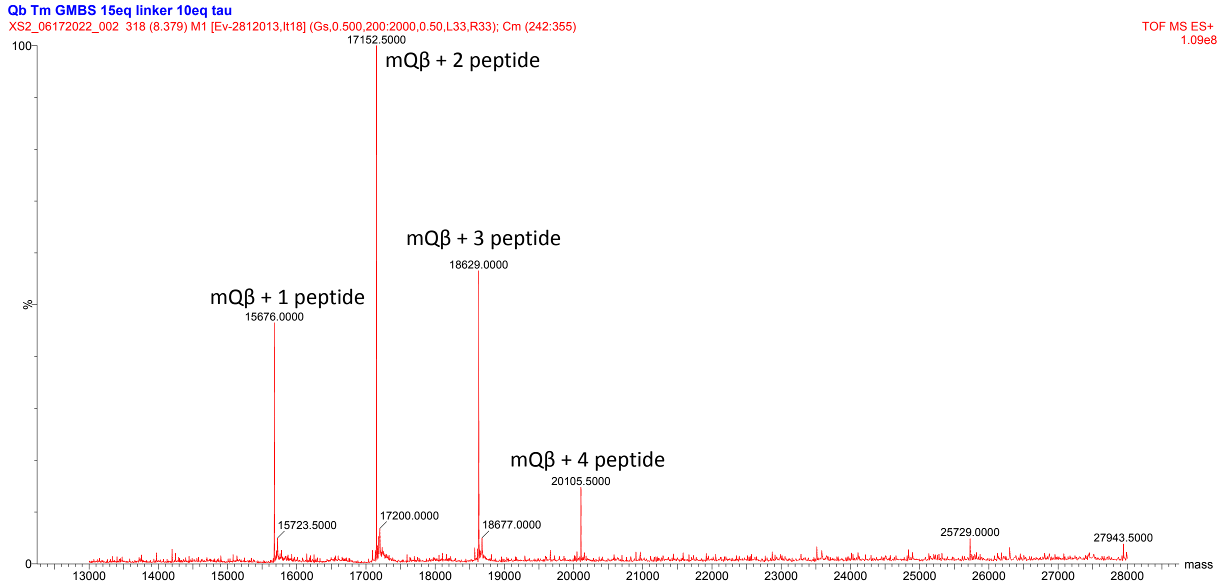

Figure S3. Deconvoluted mass spectrum from mQβ-tau synthesized using 10 eq. of GMBS and 15 eq. of peptide. Analysis of the peak intensities showed an average loading of 2.2 antigens per CP corresponding to 393 antigens per capsid.

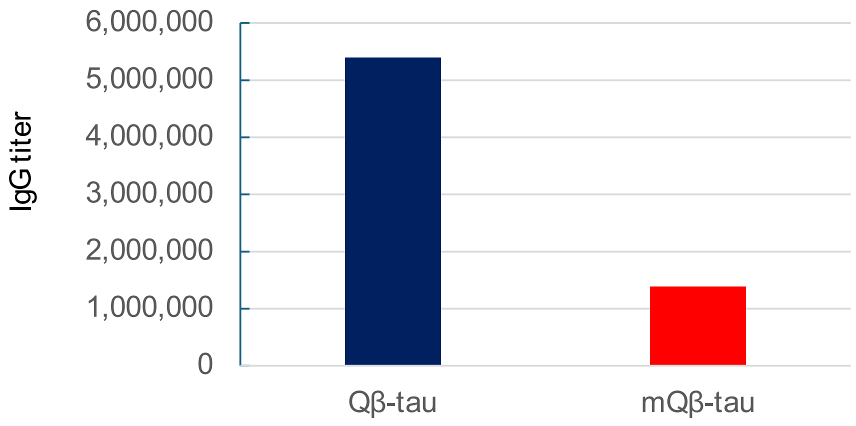

Figure S4. Immunization with mQβ-tau induced lower anti-Qβ titers as compared with Qβ-tau immunized mice. Day 35 ELISA titers against Qβ from pooled sera from mice (N = 5) immunized with either Qβ-tau or mQβ-tau. The titers were calculated as the dilution that gave an absorbance > average + 3*S.D. of a blank.

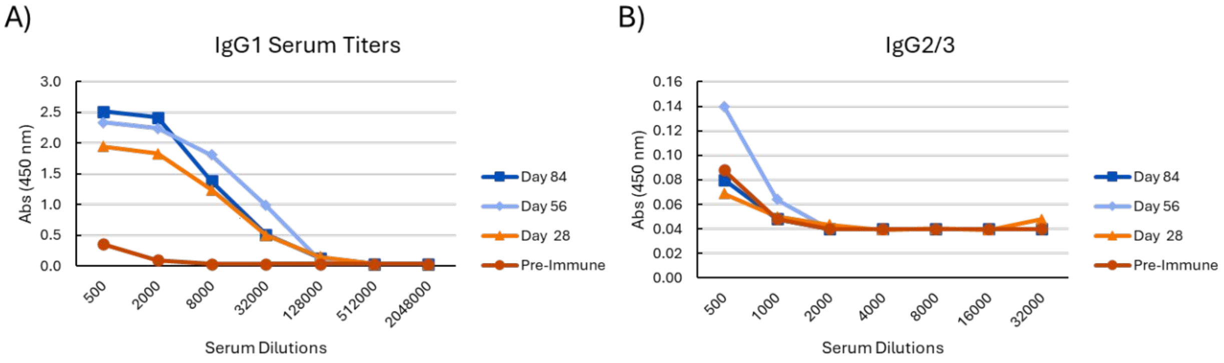

Figure S5. mQβ-tau immunization of a llama led to significant anti-tau IgG1 antibody responses. (A) IgG1 ELISA data from a llama immunized with mQβ-tau. (B) IgG2/3 ELISA data from a llama immunized with mQβ-tau.

- Ln 1: Ladder
- Ln 2: Sonication
- Ln 3: Boil
- Ln 4: Flow Thru
- Ln 5: Wash 1
- Ln 6: Wash 2
- Ln 7: Elute 1
- Ln 8: Elute 2

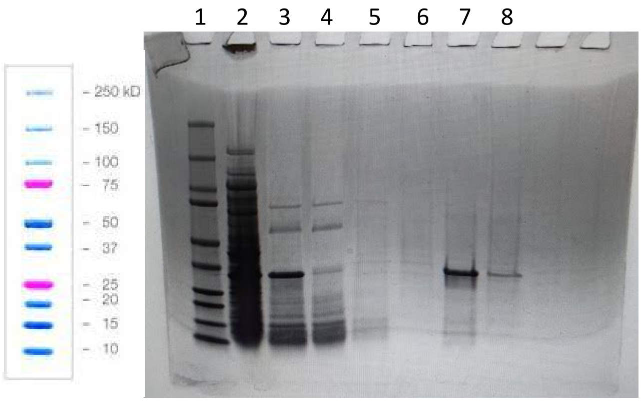

Figure S6. SDS-PAGE gel of $tau_{151\text{-}391}$-TEV-HIS purification. 10 µL of sample from each step was retained and run on a reducing SDS-PAGE gel. Protein was visualized using Coomassie Blue stain.

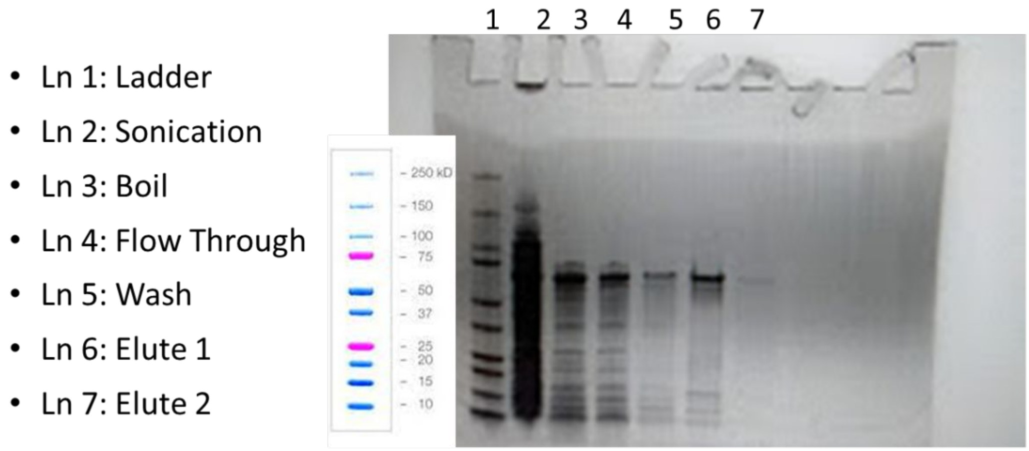

Figure S7. SDS PAGE gel of 2N4R tau Purification. 10 µL of sample from each step was retained and run on a reducing SDS-PAGE gel. Protein was visualized using Coomassie Blue stain.

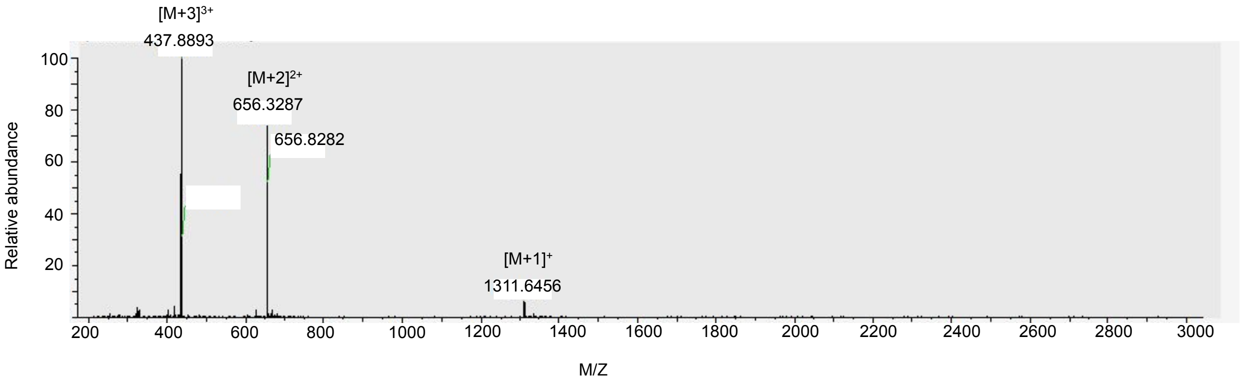

Figure S8. ESI-MS of the MTBR peptide antigen (CKDNIKHVPGGGS).

- Lane 1: Ladder
- Lane 2: mQβ-tau 101 soluble (S)
- Lane 3: mQβ-tau 102S
- Lane 4: mQβ-tau 103S
- Lane 5: mQβ-tau 104S
- Lane 6: mQβ-tau 105S
- Lane 7: mQβ-tau 106S
- Lane 8: mQβ-tau 107S
- Lane 9: mQβ-tau 108S
- Lane 10: mQβ-tau 109S

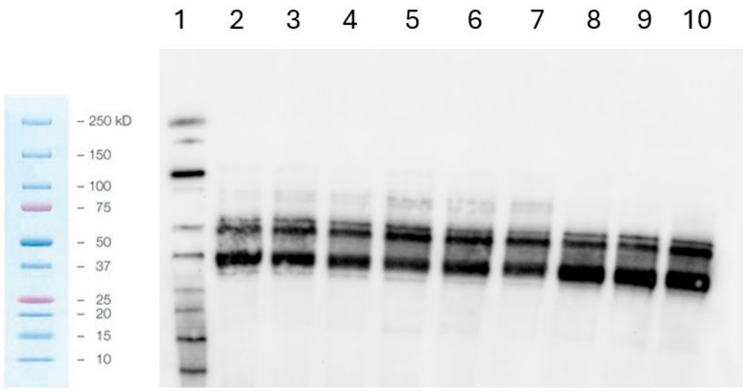

Figure S9. Representative Western blot showing Sarkosyl soluble tau protein levels isolated from brain tissues of mQβ-tau group (mouse 101 to 109). Equal volumes of each fraction were loaded per lane and separated on a 5–20% gradient SDS–polyacrylamide gel. Membranes were probed with anti-tau antibody (Tau5) followed by HRP-conjugated goat anti-mouse IgG. Immunoreactive bands were visualized by chemiluminescence.

- Lane 1: Ladder
- Lane 2: mQβ-tau 101 insoluble (I)
- Lane 3: mQβ-tau 102I
- Lane 4: mQβ-tau 103I
- Lane 5: mQβ-tau 104I
- Lane 6: mQβ-tau 105I
- Lane 7: mQβ-tau 106I
- Lane 8: mQβ-tau 107I
- Lane 9: mQβ-tau 108I
- Lane 10: mQβ-tau 109I

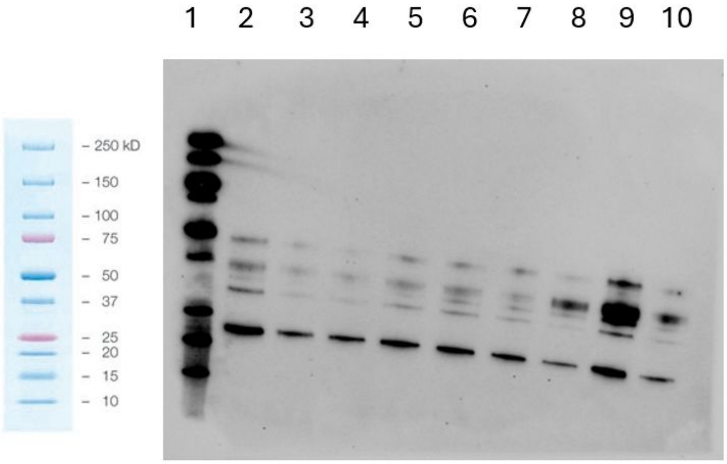

Figure S10. Representative Western blot showing Sarkosyl insoluble tau protein levels isolated from brain tissues of mQβ-tau group (mouse 101 to 109). Equal volumes of each fraction were loaded per lane and separated on a 5–20% gradient SDS–polyacrylamide gel. Membranes were probed with anti-tau antibody (Tau5) followed by HRP-conjugated goat anti-mouse IgG. Immunoreactive bands were visualized by chemiluminescence.

- Lane 1: Ladder

- Lane 2: mQβ-tau Admix 201 soluble (S)
- Lane 3: mQβ-tau Admix 202S
- Lane 4: mQβ-tau Admix 203S
- Lane 5: mQβ-tau Admix 204S
- Lane 6: mQβ-tau Admix 205S
- Lane 7: mQβ-tau Admix 206S
- Lane 8: mQβ-tau Admix 207S
- Lane 9: mQβ-tau Admix 208S
- Lane 10: mQβ-tau Admix 209S

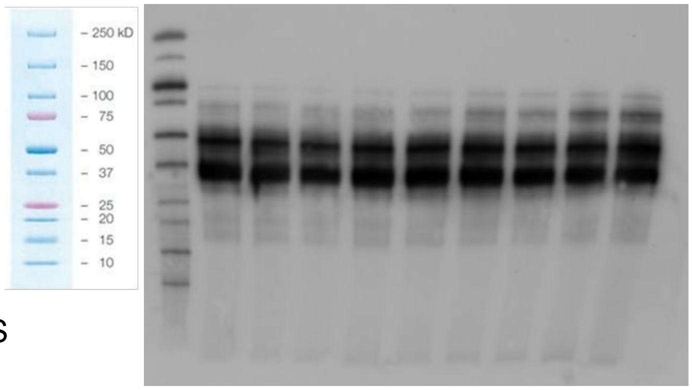

Figure S11. Representative Western blot showing Sarkosyl soluble tau protein levels isolated from brain tissues of mQβ-tau Admix group (mouse 201 to 209). Equal volumes of each fraction were loaded per lane and separated on a 5–20% gradient SDS–polyacrylamide gel. Membranes were probed with anti-tau antibody (Tau5) followed by HRP-conjugated goat anti-mouse IgG. Immunoreactive bands were visualized by chemiluminescence.

- Lane 1: Ladder
- Lane 2: mQβ-tau Admix 201 insoluble (I)
- Lane 3: mQβ-tau Admix 202I
- Lane 4: mQβ-tau Admix 203I
- Lane 5: mQβ-tau Admix 204I
- Lane 6: mQβ-tau Admix 205I
- Lane 7: mQβ-tau Admix 206I
- Lane 8: mQβ-tau Admix 207I
- Lane 9: mQβ-tau Admix 208I
- Lane 10: mQβ-tau Admix 209I

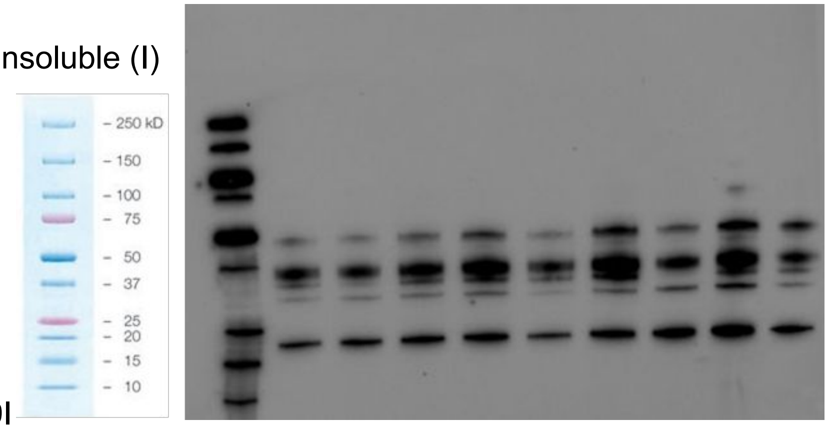

Figure S12. Representative Western blot showing Sarkosyl insoluble tau protein levels isolated from brain tissues of mQβ-tau Admix group (mouse 201 to 209). Equal volumes of each fraction were loaded per lane and separated on a 5–20% gradient SDS–polyacrylamide gel. Membranes were probed with anti-tau antibody (Tau5) followed by HRP-conjugated goat anti-mouse IgG. Immunoreactive bands were visualized by chemiluminescence.

- Lane 1: Ladder
- Lane 2: PBS 301 soluble (S)
- Lane 3: PBS 302S
- Lane 4: PBS 303S
- Lane 5: PBS 304S
- Lane 6: PBS 305S
- Lane 7: PBS 306S
- Lane 8: PBS 307S
- Lane 9: PBS 308S
- Lane 10: PBS 309S

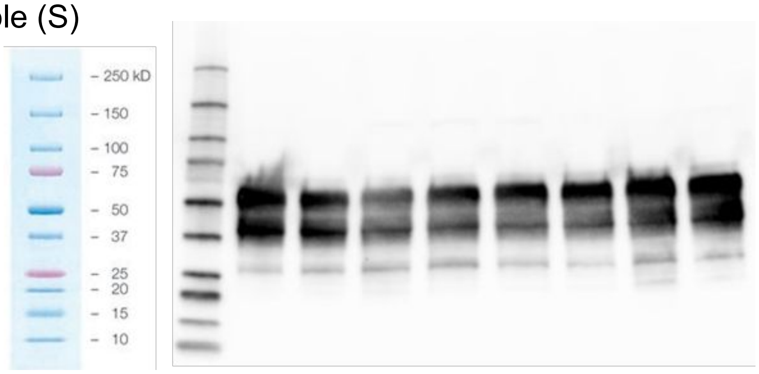

Figure S13. Representative Western blot showing Sarkosyl soluble tau protein levels isolated from brain tissues of PBS group (mouse 301 to 309). Equal volumes of each fraction were loaded per lane and separated on a 5–20% gradient SDS–polyacrylamide gel. Membranes were probed with anti-tau antibody (Tau5) followed by HRP-conjugated goat anti-mouse IgG. Immunoreactive bands were visualized by chemiluminescence.

- Lane 1: Ladder
- Lane 2: PBS 301 insoluble (I)
- Lane 3: PBS 302I
- Lane 4: PBS 303I
- Lane 5: PBS 304I
- Lane 6: PBS 305I
- Lane 7: PBS 306I
- Lane 8: PBS 307I
- Lane 9: PBS 308I
- Lane 10: PBS 309I

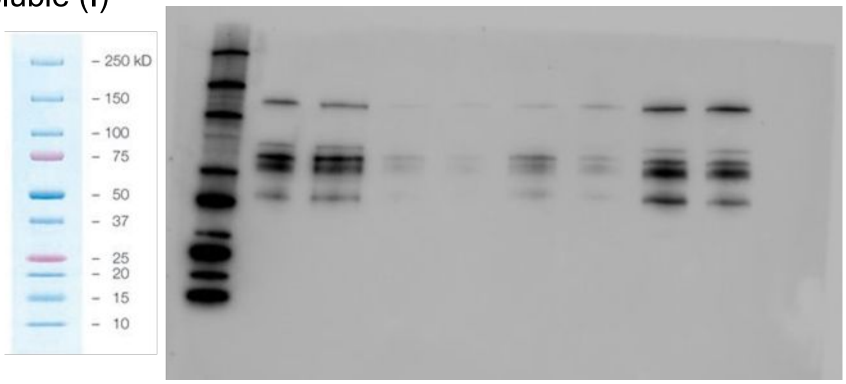

Figure S14. Representative Western blot showing Sarkosyl insoluble tau protein levels isolated from brain tissues of PBS group (mouse 301 to 309). Equal volumes of each fraction were loaded per lane and separated on a 5–20% gradient SDS–polyacrylamide gel. Membranes were probed with anti-tau antibody (Tau5) followed by HRP-conjugated goat anti-mouse IgG. Immunoreactive bands were visualized by chemiluminescence.

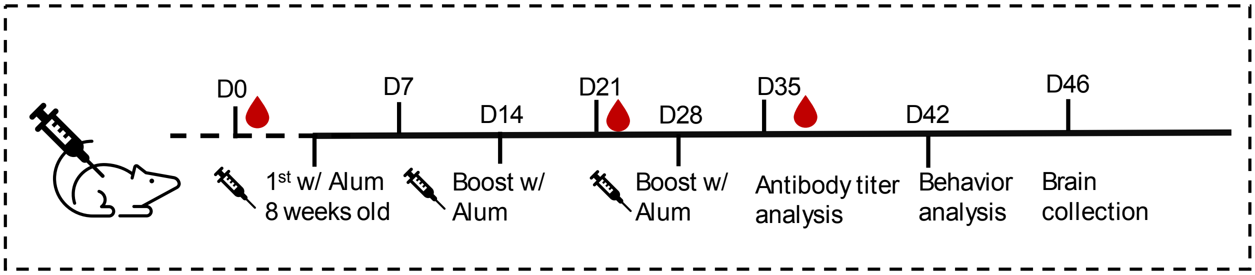

Scheme S1. Schedule for immunization and behavior studies of rTg4510 mice. Mice (female, 8 weeks old) were immunized with mQβ-tau, admixture of tau and mQβ, or PBS (N = 10 mice per group) following the one prime two boost schedule (9.6 nmol peptide per mouse per injection) over four weeks with Alum as the adjuvant. Behavior studies were conducted on day 42 after the prime immunization.
